# Neuroforecasting reveals generalizable components of choice

**DOI:** 10.1093/pnasnexus/pgaf029

**Published:** 2025-02-25

**Authors:** Alexander Genevsky, Lester C Tong, Brian Knutson

**Affiliations:** Rotterdam School of Management, Erasmus University, 3062 PA Rotterdam, The Netherlands; Department of Psychology, Stanford University, Stanford, CA 93405, USA; Department of Psychology, Stanford University, Stanford, CA 93405, USA

**Keywords:** forecasting, FMRI, brain, generalizability, representativeness

## Abstract

Accurate forecasts of population-level behavior critically inform institutional choices and public policy. While neuroforecasting research suggests that measurements of group brain activity can improve forecasting accuracy relative to behavior, less is known about how and when brain activity can effectively improve out-of-sample forecasts. We analyzed neural and behavioral data collected in two experiments to forecast choice in more vs. less demographically representative aggregate internet markets in order to test when forecasts based on brain activity generalize better than behavior. In both experiments, while the accuracy of market forecasts based on behavior varied as a function of sample representativeness, market forecasts based on brain activity remained significant regardless of sample representativeness. These findings are consistent with the notion that brain activity associated with early affective responses can generalize across individuals to index aggregate choice more broadly than downstream behavior. Thus, brain activity from limited samples may reveal generalizable components of choice that can improve market forecasts. These findings inform theory regarding which components of individual choice generalize to improve market forecasts and provide insights into mechanisms that underlie the effective application of neuroforecasting.

**Significance Statement**

The most consequential decisions in business, economics, and public policy often rely on forecasts of population-level behavior extrapolated from data collected from relatively small samples of individuals. A growing body of research in neuroforecasting suggests that neural data collected in the laboratory can improve behavioral forecasts of real-world outcomes. Little is known, however, about how neural forecasts work, how they can be improved, and how they can inform decision theory. In two experiments, individuals' neural responses to affective components of the decision-making process offered more generalizable forecasts of aggregate market demand than their behavior.

## Introduction

Accumulating neuroimaging findings suggest that neural data collected in the laboratory can add value to forecasts of real-world, population-level demand out of sample (for reviews, see Refs. 1–3). To date, however, this work has remained largely evidential, focusing on demonstrations across different market domains. Here, we focus on how neural data from relatively small laboratory samples can generalize to forecast broader aggregate behavior even when sampled self-report ratings and behavior cannot. We find mechanistic evidence that neural activity associated with early affective processes not only reliably forecasts choice out of sample, but also generalizes across diverse groups of individuals.

Forecasts of population-level behavior are typically constructed by measuring the choices of a sample of individuals and then extrapolating to the population, based on the assumption that sampled behavior will directly generalize to population behavior. Here, we test a novel account of market forecasting inspired by the emerging neuroforecasting literature, which implies that some discrete components of the decision process observable through neuroimaging may be more broadly shared across individuals than other components or even resulting choice behavior. These components should therefore support more generalizable forecasts of aggregate choice (3).

An extensive neuroeconomic literature has implicated brain activity in regions associated with affective and integrative processes in the assessment of subjective value as well as the prediction of choice in individuals (4–7). Researchers subsequently explored whether group brain activity in these regions could forecast aggregate market demand out of sample. This growing body of neuroforecasting research has demonstrated that brain data can augment, and at times surpass, forecasts based on behavior and self-report across a broad range of market domains, including but not limited to music sales (8), crowdfunding campaign success (9), advertising elasticity (10), online article sharing (11), retail sales (12, 13), and movie box-office returns (14). Together, these results provide convergent evidence that neuroforecasting can work but have yet to explain how.

These neuroforecasting findings imply that not all components supporting individual choice are equally informative in forecasting aggregate choice (3). While several decision-making processes (e.g. affective valuation, deliberative evaluation, and self-relevance) may predict and promote individual choice (5–7), they may vary in the extent to which they generalize to forecast aggregate choice (9, 10, 12, 14–20). Following this logic, identifying and extrapolating from more generalizable choice components might increase forecasting accuracy. Conversely, extrapolating from more idiosyncratic components might decrease forecasting accuracy by injecting nongeneralizable noise into aggregate forecasts.

But which components of choice should generalize most broadly? Based on neuroeconomic research, a componential decomposition of the process of decision-making can be derived from the Affect Integration Motivation (AIM) framework (21). According to this framework, choice stimuli first elicit affective responses (i.e. positive arousal and negative arousal), which are subsequently integrated through more deliberative and reflective processing (e.g. related memories, contextual considerations, and temporal extensibility), which then fuels appetitive or aversive motivational states that can potentiate observable choice behavior (e.g. approach or avoidance). Importantly, these stages of processing recruit different brain circuits. Specifically, initial affective responses elicit activity in evolutionarily conserved subcortical and cortical circuits (e.g. the Nucleus Accumbens, hereafter NAcc, and Anterior Insula, hereafter AIns). Integrative components, which are subsequently recruited and correlate with activity in the Medial PreFrontal Cortex (MPFC), may incorporate more idiosyncratic considerations into the choice process. While important for individual choice, these idiosyncratic considerations may not generalize as broadly to other individuals. Finally, observable outcomes related to self-report and choice reflect combined input from these affective and integrative processes, which are tailored to specific behavioral demands of a choice scenario, recruiting appropriate motor circuits to add a further layer of contextual specificity to the resulting action.

To explore underlying mechanisms that support neuroforecasting, we sought to connect predictions from the AIM framework with an economic random utility model of value-based choice (22–24). This model assumes that multiple sources of utility influence individual choice. For instance, in the context of neuroforecasting, these sources of utility might include initial affective responses as well as subsequent integrative valuation. The combined input of these factors then might predict an option's overall utility and subsequent individual choice. Formally, a final utility (*U*) for any individual (i) and option (o) can be described as a function of a shared component of value common across individuals (*V*_ao_) and an idiosyncratic component of value unique to each individual (*V*_io_; specifically, *U*_io_ = *V*_ao_ + *V*_io_).

Combining the neuroeconomic AIM framework and the economic random utility model, individual choice might therefore include both shared affective components more common across individuals (*V*_a_) as well as idiosyncratic integrative components more unique to each individual (*V*_i_). This combined model can be contrasted with more conventional behavioral models based on observed choice. Generalized to forecasts of aggregate choice, the combined model implies two novel predictions. First, in smaller samples, individual value components (*V*_i_) might introduce noise into aggregate models, reducing the accuracy of forecasts. In these cases, including shared value components (*V*_a_) might improve forecasts. Second, nonrepresentative samples with idiosyncratic value components that differ from those of a population might bias forecasts. Thus, forecasts based on shared value components may generalize more broadly than those based on idiosyncratic value components or observed choice.

**Fig. 1.**
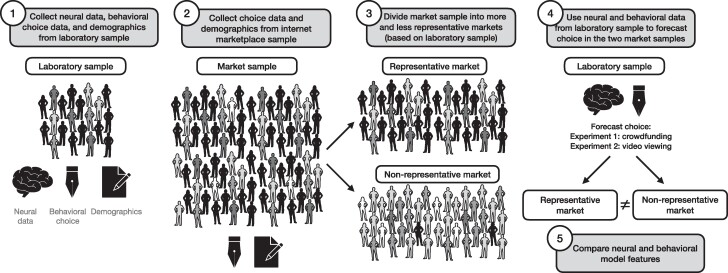
(1) Brain, behavioral, and demographics data were collected in laboratory samples. (2). Behavior and demographics data were collected in constructed internet markets. (3). Internet markets were divided into more and less representative markets based on demographic match to the laboratory samples. (4) Neural and behavioral data in laboratory samples were used to forecast behavioral data in more and less representative market samples. (5) Neural and behavioral model features were contrasted in more and less representative market samples.

In two experiments, neuroimaging and choice data collected in independent neuroforecasting studies (9, 20) were used to forecast aggregate preference in newly collected internet market samples that systematically varied with respect to representativeness. Brain activity and behavioral choice data from the laboratory samples were then used to forecast demand as a function of demographic match with the internet samples (for a design overview, see Fig. 1). We then tested whether neural predictors of choice could generalize more broadly than choice behavior across samples varying in representativeness to better understand the mechanisms underlying the generality of neuroforecasts. Additionally, we tested the robustness of neurally derived forecasts across sample sizes and demonstrated that reliable forecasts could be obtained from relatively small laboratory samples to address perceived cost obstacles to the practical application of neuroforecasting methods (25).

## Results

### Total market analyses

We first tested for associations of laboratory sample measures (both behavior and neural) with the choices of substantially larger internet markets (including all online subjects, *n* = 2,956; Table 1). Considering only behavioral predictors, in the crowdfunding experiment, laboratory sample choices were directionally but not significantly associated with internet sample funding choices (*t* = 1.933, *P* = 0.062), and in the video-viewing experiment, laboratory sample choices were again not significantly associated with internet sample choices (*t* = −0.44, *P* = 0.661). These results are consistent with positive but nonsignificant associations observed between behavioral preferences in the internet samples and the real-world markets used in the original crowdfunding and video-viewing studies, possibly attributable to sample, design, and temporal differences across paradigms (experiment 1: *r* = 0.12; experiment 2: *r* = 0.24) (9, 20).

**Table 1.** Models forecasting aggregate behavior across the entire internet market in crowdfunding (1) and video-viewing (2) experiments.

	Crowdfunding study Laboratory *n* = 37 Out-of-sample *n* = 2956			Video-viewing study Laboratory *n* = 40 Out-of-sample *n* = 992		
	Behavior	Neural	Combined	Behavior	Neural	Combined
Lab sample behavior	0.315 (0.163)		0.252 (0.153)	−0.071 (0.182)		−0.105 (0.168)
NAcc Activity		0.664** (0.218)	0.644** (0.213)		0.512** (0.181)	0.520** (0.183)
MPFC Activity		−0.274 (0.218)	−0.330 (0.216)		−0.189 (0.181)	−0.204 (0.185)
*R* ^2^	0.099	0.252	0.311	0.005	0.216	0.227
AIC	103.40	98.67	97.75	95.63	90.01	91.56

Statistics are standardized coefficients with SEs in parentheses. *p < 0.05; **p < 0.01; ***p < 0.001.

Next, in models including activity in predicted neural regions of interest (centered in the NAcc and MPFC), only laboratory sample NAcc activity was significantly associated with aggregate choices in both the crowdfunding and video-viewing internet samples (crowdfunding: *t* = 3.043, *P* = 0.004; video viewing: *t* = 4.09, *P* < 0.001). Despite predicting trial-by-trial choice within individuals, laboratory sample MPFC activity was not associated with aggregate sample choice in either internet sample (experiment 1: *t* = −1.257, *P* = 0.218; experiment 2: *t* = −1.48, *P* = 0.145). In a final combined model, including both behavioral and neural data, only laboratory sample NAcc activity remained significantly associated with internet sample aggregate choice (experiment 1: *t* = 3.02, *P* = 0.005; experiment 2 *t* = 4.12, *P* < 0.001; Table 1). Together, these analyses suggest that only brain activity (i.e. in the NAcc) of the scanned laboratory samples significantly forecasts aggregate choice in internet samples across both experiments.

### Representativeness analyses

To identify the most generalizable components of choice, we next examined the impact of sample representativeness by repeating the same analyses on the most vs. least representative internet sample quartiles (determined by similarity across six common demographic variables, see Materials and methods for additional details). For the crowdfunding experiment, in the most representative internet sample, both laboratory sample choices (*t* = 2.70, *P* = 0.011) and NAcc activity (*t* = 2.81, *P* = 0.008) were significantly associated with internet sample choices. In the nonrepresentative internet sample, however, only NAcc activity was significantly associated with internet sample choices (*t* = 2.84, *P* = 0.007; Table 2). Robustness checks revealed similar results when also including activity from the AIns in the models (Table S3), or when dividing the internet sample using a median split (Table S1). This same pattern of results was also observed for the video-viewing experiment (Tables 3 and S2).

**Table 2.** Models forecasting aggregate choice (to fund or not) in the most representative and least representative crowdfunding markets (experiment 1).

	Most representative quartile			Least representative quartile		
	Behavior	Neural	Combined	Behavior	Neural	Combined
Lab sample behavior	0.441** (0.154)		0.400* (0.148)	0.275 (0.165)		0.214 (0.157)
NAcc activity		0.612* (0.225)	0.579** (0.206)		0.638** (0.221)	0.621** (0.219)
MPFC activity		−0.265 (0.225)	−0.353 (0.208)		−0.270 (0.221)	−0.317 (0.221)
*R* ^2^	0.194	0.209	0.356	0.076	0.231	0.273
AIC	99.38	100.69	95.28	104.32	99.71	99.68

Statistics are standardized coefficients with SEs in parentheses. MPFC, Medial PreFrontal Cortex; NAcc, Nucleus Accumbens. *p < 0.05; **p < 0.01; ***p < 0.001.

**Table 3.** Models forecasting aggregate choice (to watch or not) in the most representative and least representative video-viewing markets (experiment 2).

	Most representative quartile			Least representative quartile		
	Behavior	Neural	Combined	Behavior	Neural	Combined
Lab sample behavior	0.065 (0.182)		0.027 (0.172)	−0.189 (0.179)		−0.216 (0.163)
Nucleus accumbens		0.473* (0.184)	0.471* (0.188)		0.512** (0.180)	0.530** (0.178)
MPFC		−0.269 (0.184)	−0.265 (0.189)		−0.115 (0.180)	−0.145 (0.179)
*R* ^2^	0.004	0.189	0.189	0.036	0.226	0.272
AIC	95.66	91.11	93.08	94.63	89.60	89.64

Statistics are standardized coefficients with SEs in parentheses. MPFC, Medial PreFrontal Cortex; NAcc, Nucleus Accumbens. *p < 0.05; **p < 0.01; ***p < 0.001.

**Fig. 2.**
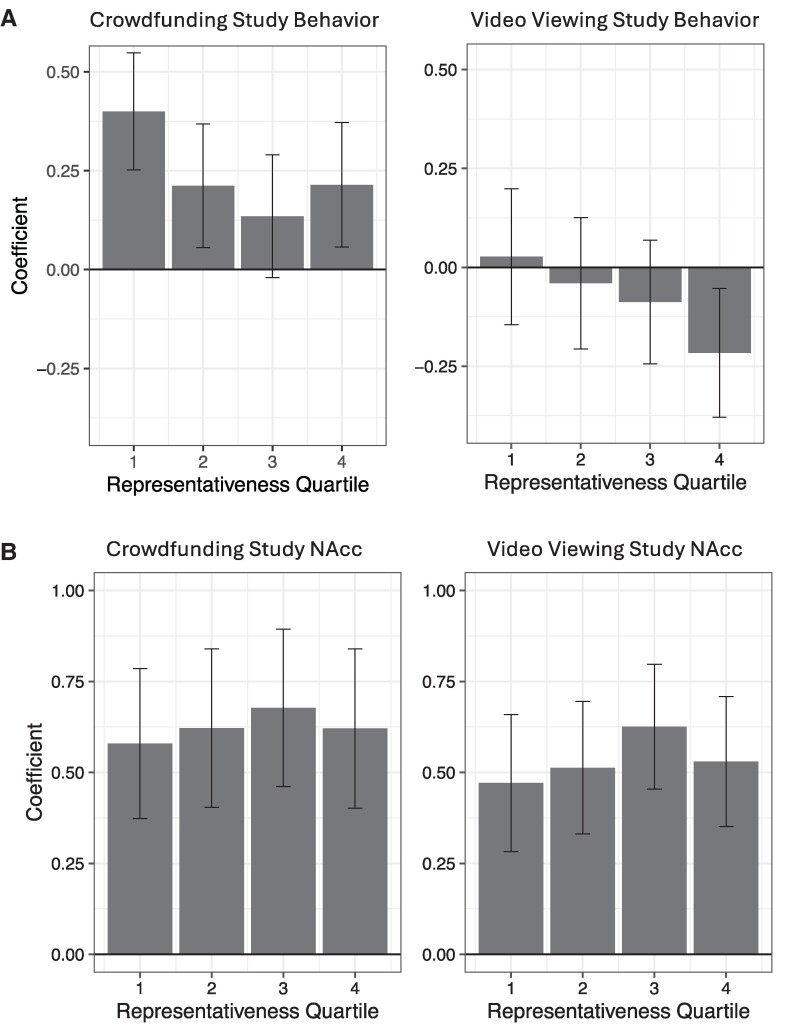
Coefficients from behavioral and neural models forecasting aggregate choice as a function of market demographic similarity. Displayed from the most representative (left; 1) to the least representative (right; 4) quartiles. Behavioral coefficients increased as a function of demographic match (A), but brain (i.e. NAcc) coefficients did not (B).

Across experiments, laboratory sample choice forecasts of aggregate internet sample choice interacted with quartile demographic match (*t* = −2.57, *P* = 0.012), suggesting that the strength of the association between laboratory sample choices and internet sample choices depended on representativeness. Importantly, however, NAcc activity forecasts did not significantly interact with demographic match (*t* = −0.587, *P* = 0.558), suggesting that neural forecasts depended less on sample representativeness, and so might support broader generalization. Plots of the behavioral coefficients across representativeness quartiles confirmed that the association of laboratory choice with aggregate choice diminished with decreasing demographic match, while the association of laboratory NAcc activity with aggregate choice remained constant regardless of demographic match (Fig. 2 and Table S4).

Analyses within the internet samples indicated that decreasing demographic match across quartiles was associated with reduced similarity in behavioral choice (Fig. S2). This pattern validated the proposed association of demographic match with behavioral preferences, consistent with the notion that reduced representativeness might constrain behavioral forecasts. Forecasts based on neural activity, however, did not vary as a function of demographic match. Bootstrapped analyses further tested the relative impact of representativeness on behavioral and neural forecasts of aggregate choice. In 96.7% of analytic iterations in the crowdfunding experiment and 97.2% of analytic iterations in the video-viewing experiment, behavioral coefficients were more diminished by decreases in representativeness than neural coefficients (*P* = 0.033), consistent with broader generalizability of neural forecasts of aggregate choice across different internet samples.

Analyses within the laboratory sample further explored the generalizability of neural activity associated with affective and integrative neural processes by comparing the similarity of responses in the NAcc vs. MPFC to study stimuli across individuals (Fig. S3). Interclass correlations indicated that only NAcc activity was significantly correlated across individuals in the crowdfunding experiment (ICC = 0.441, *F* = 1.82, *P* = 0.004), while MPFC activity was not (MPFC: ICC = 0.273, *F* = 1.38, *P* = 0.080). Analysis of the video-viewing experiment revealed a similar pattern of results, since NAcc activity was significantly correlated across individuals, while MPFC activity was not (NAcc: ICC = 0.408, *F* = 1.70, *P* = 0.009; MPFC: ICC = 0.198, *F* = 1.25, *P* = 0.163). These analyses further support the idea that neuroforecasting relies on decision processes that are shared across individuals.

### Sample-size robustness analyses

While neuroforecasting data might add value to conventional behavioral studies, they might also cost more since their collection requires specialized equipment and expertise. These constraints consequently raise questions about how many subjects are required for neuroforecasting studies to yield generalizable findings. To address this question, we conducted bootstrapped analyses that varied the number of individuals used to forecast aggregate choice.

**Fig. 3.**
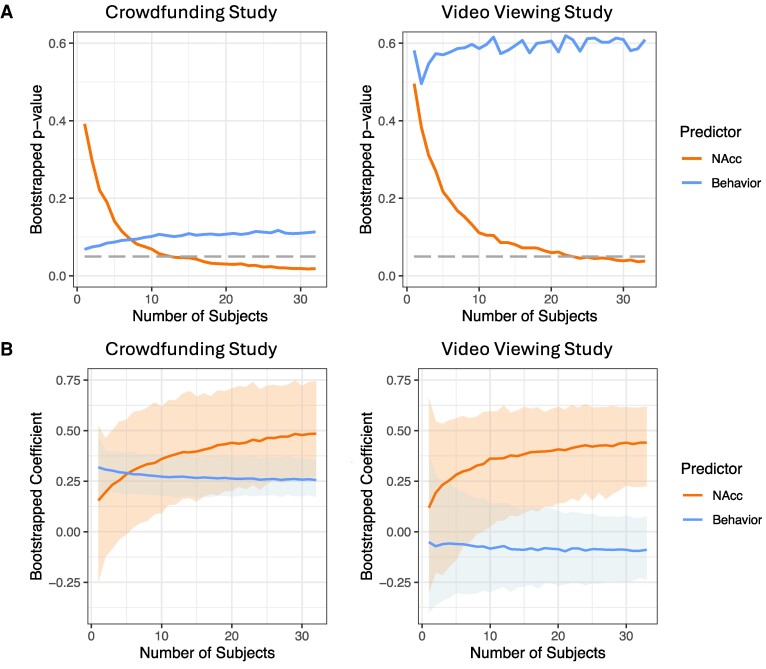
Bootstrapped analyses of the impact of sample size on forecasting. Estimates of A) *P*-values and B) coefficients for brain (NAcc activity) vs. behavioral (choice) forecasts of market-level preferences as a function of the number of sampled subjects.

In the crowdfunding experiment, as the number of subjects increased, the median estimate for NAcc activity dropped sharply, falling below the *P* = 0.05 threshold at 14 subjects and remaining consistently below this threshold through 32 subjects (Fig. 3A). Estimates for behavior, however, remained nonsignificant as the sample size increased and did not descend below the *P* = 0.05 threshold. Analyses of coefficient magnitudes for neural vs. behavioral measures revealed complementary trends. Specifically, as sample size increased, the NAcc coefficient rose, approaching asymptote (Fig. 3B), whereas the behavioral coefficient remained relatively flat. Analyses of data from the video-viewing experiment revealed similar patterns (Fig. 3). As the sample size increased, the NAcc significance estimate dropped below the *P* = 0.05 threshold at 23 subjects, while the behavioral significance estimate remained nonsignificant over the range of iterations tested.

Together, these findings suggest that relatively small sample sizes can support forecasts of aggregate choice based on NAcc activity (see Fig. S1 for MPFC activity, which showed an intermediate pattern). In the crowdfunding and video-viewing markets examined, a sample size of 20–25 subjects seemed sufficient to support aggregate forecasts with brain data. The applicability of this pattern of findings to other types of markets remains to be explored.

## Discussion

Across two studies, forecasts of aggregate choice based on neural activity generalized more broadly than forecasts based on behavioral data. These findings suggest that while both generalizable affective components and idiosyncratic integrative components predict individual choice, generalizable affective components can forecast aggregate choice even when behavioral measures cannot. In both experiments, increased demographic match of laboratory samples to internet samples improved behavioral forecasts. Within internet markets, demographic match was also associated with more similar behavioral choices, supporting conventional wisdom that the behavior of representative samples should more accurately forecast population behavior. In contrast, neural forecasts of aggregate internet sample choice remained significant despite decreases in demographic match. While counterintuitive, the superior generalizability of brain activity vs. behavioral choice suggests that early anticipatory affective responses might generalize more broadly than subsequent integrative or behavioral responses (21).

Conceptually, these findings link theories derived from decision neuroscience (i.e. the AIM framework) and economics (i.e. random utility models) to explain previous empirical findings and elucidate how neural data can improve forecasts of aggregate behavior. Conventional theoretical approaches in psychology and economics (e.g. expected value theory) might imply that the same neural activity that predicts individual choice should also forecast aggregate choice (perhaps with some loss due to added noise; 3). Instead, this novel evidence supports and extends a random utility-based account of neuroforecasting (23) in which some components of value are more broadly shared than others (21). Specifically, initial affective responses (indexed by NAcc activity) represent a more commonly shared component of the decision-making process than later integrative neural responses (indexed by MPFC activity) or even final choice behavior itself. Further, analyses revealed that within laboratory samples, individuals' NAcc responses were more correlated than their MPFC responses. While both affective and integrative processes critically contribute to individual choice, integration may incorporate more idiosyncratic factors, and so reflect less of the aggregate choices of others.

These findings have implications for which types of markets might benefit most from neuroforecasting. In this research, the most generalizable neural signals came from circuits associated with affective processing. Thus, forecasts in markets for approaching positive outcomes (e.g. hedonic ventures and experiences) might prominently recruit circuits implicated in positive anticipatory affect (e.g. the NAcc), and so might benefit most from the application of neuroforecasting measures. In turn, identification of generalizable choice components can imply levers for intervention. For example, microloan appeals that feature a positive face, which can increase NAcc activity, are more likely to receive funding from individuals as well as internet markets (18).

Other markets, however, might more prominently recruit other choice components. For instance, according to a “market matching” account (3), markets for avoiding negative outcomes (e.g. purchasing insurance) might more prominently recruit circuits implicated in negative anticipatory affect (e.g. the AIns). Further, other markets focusing on time or social considerations might more prominently recruit integrative circuits (e.g. the MPFC) (26). Future research might profitably examine how broadly affective primacy vs. market matching accounts extends to different markets. Additionally, behavioral manipulations that emphasize different choice components (e.g. encouraging reliance on affective processes, or minimizing integration) might change choice predictably in the laboratory and impact forecasting performance.

This work also addresses a primary obstacle to the practical adoption of neuroimaging methods in business, communications, and public policy involving perceptions of prohibitive cost. In both experiments, results indicated that a reasonably small sample (e.g. *n* < 30) was sufficient to derive stable forecasts from neural data. These findings are consistent with a recent review of sample sizes implemented in the existing Functional Magnetic Resonance Imaging (FMRI) neuroforecasting studies to date, which tended to average around 30 subjects (3). Interesting questions about whether some individuals reliably show more diagnostic brain activity and whether they can be identified prior to scanning remain to be explored and might further reduce costs associated with neuroforecasting.

These combined findings suggest that when a representative sample is available, the addition of neural data might still account for added variance in forecasts of aggregate choice. Thus, brain measures may complement behavioral measures to improve representative forecasts of aggregate behavior. Even when a representative sample is not available (e.g. due to lack of accessibility, unwillingness to participate, or missing information about the target audience), brain measures might still effectively forecast aggregate choice.

In summary, this work suggests that one mechanism underlying successful neuroforecasting involves generalizable components of choice revealed by neural measures. In contrast, self-reported responses and observed behavioral choices incorporate idiosyncratic preferences which can contribute to accurate predictions of individual behavior but diminish the accuracy of aggregate forecasts. Together, these findings illuminate how brain measures may reveal seeds of choice that can generalize to forecast market behavior—even when behavioral measures cannot.

## Materials and methods

### Subjects

In the crowdfunding experiment (experiment 1), 37 healthy right-handed human adults participated in the neuroimaging phase (17 females; mean age, 23.57). In the video-viewing experiment (experiment 2), 40 subjects participated in the neuroimaging phase (25 females; mean age, 25.28; see Appendix A for full subject descriptive statistics). Subjects were screened for psychotropic drug use, substance use, and a history of neurological disorders, as well as for typical magnetic resonance exclusions (e.g. metal in the body). All procedures were approved by the Stanford University IRB. In both experiments, subjects were excluded for excessive head movement during scanning (i.e. more than four instances >6 mm or two voxel sides from one volume acquisition to the next), and for incomplete demographic data (required for demographic matching), leaving samples of 32 and 33 subjects, respectively, for analysis. All subjects received detailed information regarding their rights and the protections of their data before consenting to participate.

### Crowdfunding experiment (experiment 1)

#### Crowdfunding design

In the neuroimaging phase of the crowdfunding experiment, subjects were presented with text and images associated with 36 crowdfunding projects selected from kickstarter.com while being scanned with FMRI. On each trial, subjects were asked to make binary incentive-compatible decisions about whether or not to fund a project (for more details, see Ref. 9). For the internet market sample in the crowdfunding experiment, 3,000 online subjects were recruited to make similar preference judgments regarding these same projects. Demographic variables (i.e. age, sex, education, ethnicity, socioeconomic status, marital status, and employment status) collected from both laboratory and internet subjects were then used to partition the sample into submarkets that varied with respect to representativeness (detailed below). Finally, laboratory subjects' behavioral responses and neural activity were used to forecast the preferences of more and less representative internet samples.

#### Crowdfunding scanning task

While being scanned, subjects made incentive-compatible funding choices regarding 36 crowdfunding film projects selected from kickstarter.com (9). On each trial, subjects viewed a photographic image from each online funding page (2 s), followed by text briefly describing the project (6 s). Subjects were then asked to make a binary “Yes/No” decision regarding whether they would like to fund the project (4 s). Laterally counterbalanced Yes and No options were presented on either side of the screen, and choices were made using corresponding buttons on a handheld button box. Finally, subjects viewed a centrally presented fixation cross for a variable intertrial interval before the next trial began (2–6 s). Total trial duration (including the intertrial interval) thus averaged 16 s (range, 14–18 s). Overall, subjects evaluated 36 unique funding requests. Subjects were informed that one trial would be randomly selected to count for real at the conclusion of the experiment. If subjects had agreed to fund the randomly selected appeal, that amount was subtracted from their payment and contributed online to the appropriate project; otherwise, subjects retained their full endowment (for task design schematic and stimuli, see Appendix B).

#### Crowdfunding market choice task

Individuals were sampled on the internet (*n* = 2956, see Appendix A for descriptive statistics) via an online subject pool (Amazon Mechanical Turk) to complete an online crowdfunding preference task (Appendix B). On each trial, subjects were presented with two documentary film projects used in the neuroimaging study and asked to choose which they preferred. For each film project, subjects viewed the same image and descriptive text as subjects in the neuroimaging study. For each subject, the 36 projects were randomly paired and presented together such that each project was presented only once. Finally, the aggregate preference for each project was calculated by adding the number of times each project was selected across all subjects. The randomization procedure and large sample size ensured that this value represented the group's relative preference for each project relative to all other presented projects. In analyses contrasting samples of different representativeness (detailed below), aggregate preferences were calculated in this manner independently for each sample. After the crowdfunding preference task, subjects completed a demographic questionnaire (Appendix B).

### Video-viewing experiment (experiment 2)

#### Video-viewing design

In the neuroimaging phase of the video-viewing experiment, 40 subjects were presented with 32 videos selected from youtube.com while being scanned with FMRI. In the internet phase of the study, online subjects (*n* = 1,000) were recruited to make similar preference judgments regarding these same videos. As in the crowdfunding experiment, demographic variables collected from both laboratory and internet subjects were used to create samples of varying representativeness. Laboratory subjects' behavioral responses and neural activity were then used to forecast the preferences of the larger internet samples.

Videos included clips selected from the popular youtube.com science channels “Discovery” and “Animal Planet” lasting from 54 to 172 s (for full list, see Appendix C). These videos were culled from a larger database of 2,950 videos whose thumbnails had previously been effectively normed with a larger online (MTurk) sample in a pilot study (20). Video stimulus sampling was designed to maximize variance in aggregate video-view duration (calculated as a percentage of the total video length; view percentage), as well as affective ratings of their thumbnail images (i.e. high vs. low arousal and high vs. low valence).

#### Video-viewing scanning task

In a preliminary task, laboratory sample subjects saw video thumbnail images and indicated whether they wanted to watch the video by pressing a button box to choose the corresponding option on the right or the left, respectively (4 s). Placement of accept (vs. reject) response buttons was laterally randomized across trials. During each trial of the subsequent video-viewing task, a centrally displayed video began playing, which was then followed by a gray square that randomly appeared after 4–8 s to the right or left side of the video. Subjects could then choose to skip the rest of the video at any subsequent point by pressing a button corresponding to the position of the gray square. Subjects completed 32 trials of the video-viewing task, in 4 runs of 8 trials each (for study design schematic, see Appendix C). To control stimulus content, all subjects had to watch at least the first 4, 6, or 8 s of all 32 videos (forced view time was randomly varied to ensure nonpredictability). Trials were presented in one of two pseudorandom orders (i.e. either a forward- or reverse-ordered sequence).

#### Video-viewing market choice task

Individuals were recruited on the internet (*n* = 992, see Appendix A for descriptive statistics) via an online subject pool (Amazon Mechanical Turk) to complete an online video-viewing preference task (Appendix B). On each of 32 trials, subjects saw one of the videos used in the neuroimaging study. After watching the first 15 s of each video, subjects made a binary decision (i.e. Yes or No) about whether they would be interested in continuing to watch the video. Regardless of the registered response, subjects then proceeded to the next trial. All 32 videos were presented in a random order to each subject. Aggregate preference for each video was calculated by summing the number of choices to continue watching each video across all subjects. In analyses comparing samples of varying representativeness, choices to watch or not were independently aggregated for each sample. After the video-viewing task, subjects completed a short demographic questionnaire. While the internet market and laboratory tasks in both experiments were designed to approximate the real-world choice scenarios as closely as possible, preference elicitation varied in some respects due to logistical, temporal, and financial constraints. Importantly, however, all analyses contrasted neural and behavioral forecasts that extended to the same elicitation procedures.

#### Market creation

In both experiments, samples of varying representativeness were created by dividing the internet samples (*n* = 2,956 and *n* = 992, respectively; Appendix A) into four equally sized samples that varied as a function of multivariate demographic match to the laboratory samples. To quantify demographic match, the Mahalanobis distance (27, 28) was calculated for each member of the laboratory and internet samples based on a vector of seven demographic variables (i.e. age, gender, education, socioeconomic status, education, marital status, and employment status). For each individual in the laboratory sample, the Mahalanobis distance was calculated as the square root of the sum of squared differences between the point and the distribution, weighted by the inverse covariance matrix of the distribution. Subsequently, online subjects were rank ordered based upon their demographic distance to the neuroimaging sample. Finally, to create subsamples for calculating aggregate preference, a quartile split on the Mahalanobis distance metric divided the larger internet samples into subsets that varied with respect to their demographic match to the neuroimaging sample. The Mahalanobis distance accounts for covariation between variables, allowing for a more accurate assessment of distances between observations and a distribution, particularly where variables can be correlated. The Mahalanobis distance is often applied in multivariate statistics to compare the similarity of groups of observations. For this reason, it offers a particularly useful tool for researchers in domains where individual-level variables are often both multidimensional and correlated.

#### FMRI acquisition and analyses

In the neuroimaging experiments, images were acquired with a 3.0-T General Electric MRI scanner using a 32-channel head coil. Forty-six 2.9-mm thick slices (in-plane resolution, 2.9 mm cubic; no gap; interleaved acquisition) extended axially from the mid-pons to the crown of the skull, providing whole-brain coverage and good spatial resolution of mesolimbic regions of interest (e.g. midbrain, NAcc, and MPFC). Whole-brain functional scans were acquired with a T_2_*-weighted gradient echo pulse sequence (TR = 2 s; TE = 24 ms; flip angle, 77°). High-resolution structural scans were acquired with a T_1_-weighted pulse sequence (TR = 7.2 ms; TE = 2.8 ms; flip angle, 12°) after functional scans to facilitate their localization and coregistration.

Primary analyses were conducted using a priori defined volumes of interest (VOIs) derived from previous work on the AIM framework (21) and neuroforecasting (8, 9, 11, 17, 18). Based on this work, predicted regions included those associated with anticipatory positive affect (i.e. NAcc) (29) and value integration (i.e. MPFC) (5, 21, 30). Spherical VOIs (8 mm diameter) were centered bilaterally on foci in the NAcc (Talairach coordinates: ±10, 12, −2) and MPFC (±4, 45, 0). FMRI activity (percentage signal change) was first averaged within each VOI over the first two image volume acquisitions (i.e. 4 s) of stimulus presentation (lagged by 4–6 s to account for the hemodynamic response function), next averaged bilaterally, and then extracted for subsequent analyses. These predicted VOIs also showed high overlap with meta-analytic map regions with activity associated with their names derived from the Neurosynth database (31) (Appendix D).

For bootstrapped analyses, a distribution of estimates for the coefficients of laboratory choice and NAcc activity was derived over 10,000 iterations using samples drawn randomly from the original data with replacement. On each iteration, models regressed behavioral and neural predictors on aggregate choice. We then calculated the difference in coefficient estimates between identical models applied to the most and least representative sample quartiles, representing the magnitude of the impact of representativeness for the predictors on each iteration. Next, we assessed the proportion of iterations in which the behavioral estimate differences were greater than the median of the NAcc estimate differences. In the sample-size analysis, samples ranging from 32 subjects to 1 subject (i.e. the video-viewing experiment included 33 subjects), 1,000 bootstrapped samples were drawn from the original data with replacement. After each resampling iteration, we regressed internet sample choices on behavioral and neural variables drawn from corresponding laboratory samples.

## Supplementary Material

pgaf029_Supplementary_Data

## Data Availability

Anonymized data are publicly available on an online repository (32). Study materials are available in the Supplementary material.
