# Supplementary material for: Neuroforecasting reveals generalizable components of choice
Source: PNAS Nexus. 2025 Feb 25;4(2):pgaf029. doi: 10.1093/pnasnexus/pgaf029 (PMC11852258; doi:10.1093/pnasnexus/pgaf029)

**
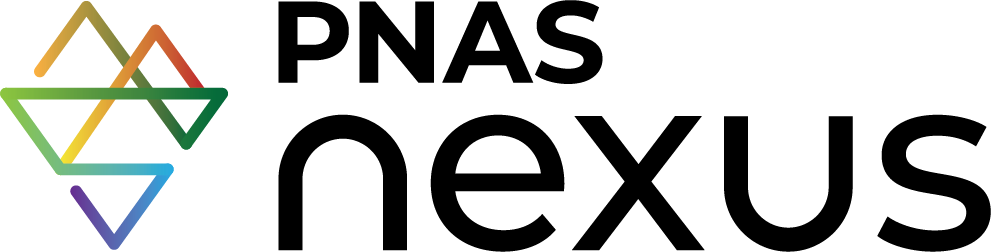
**

**Supporting Information for**

Neuroforecasting reveals generalizable components of choice

Alexander Genevsky^1*^, Lester Tong^2^, and Brian Knutson^2^

^1^ Rotterdam School of Management, Erasmus University

^2^ Department of Psychology, Stanford University

Corresponding author:

Alexander Genevsky

Department of Marketing

Rotterdam School of Management, Erasmus University Rotterdam

3062 PA, The Netherlands.

**Email:** [genevsky@rsm.nl](mailto:genevsky@rsm.nl)

**This PDF file includes:**

Figures S1 to S3

Tables S1 to S4

Supporting text Appendices A-D

Fig. S1. Bootstrapped analyses of the impact of sample size on forecasting. Estimates of A) p-values and B) coefficients for brain (NAcc and MPFC activity) versus behavioral (choice) forecasts of market-level preferences as a function of the number of sampled subjects.

A.

B.

**Fig. S2. Correlations of behavior across representative quartiles.** Across two experiments, **c**orrelations of behavioral preferences decrease as the demographic similarity between quartiles decreases. Numerals along the x and y axes indicated quartiles varying in demographic similarity to neuroimaging samples (i.e., q1: most similar; q4 least similar).


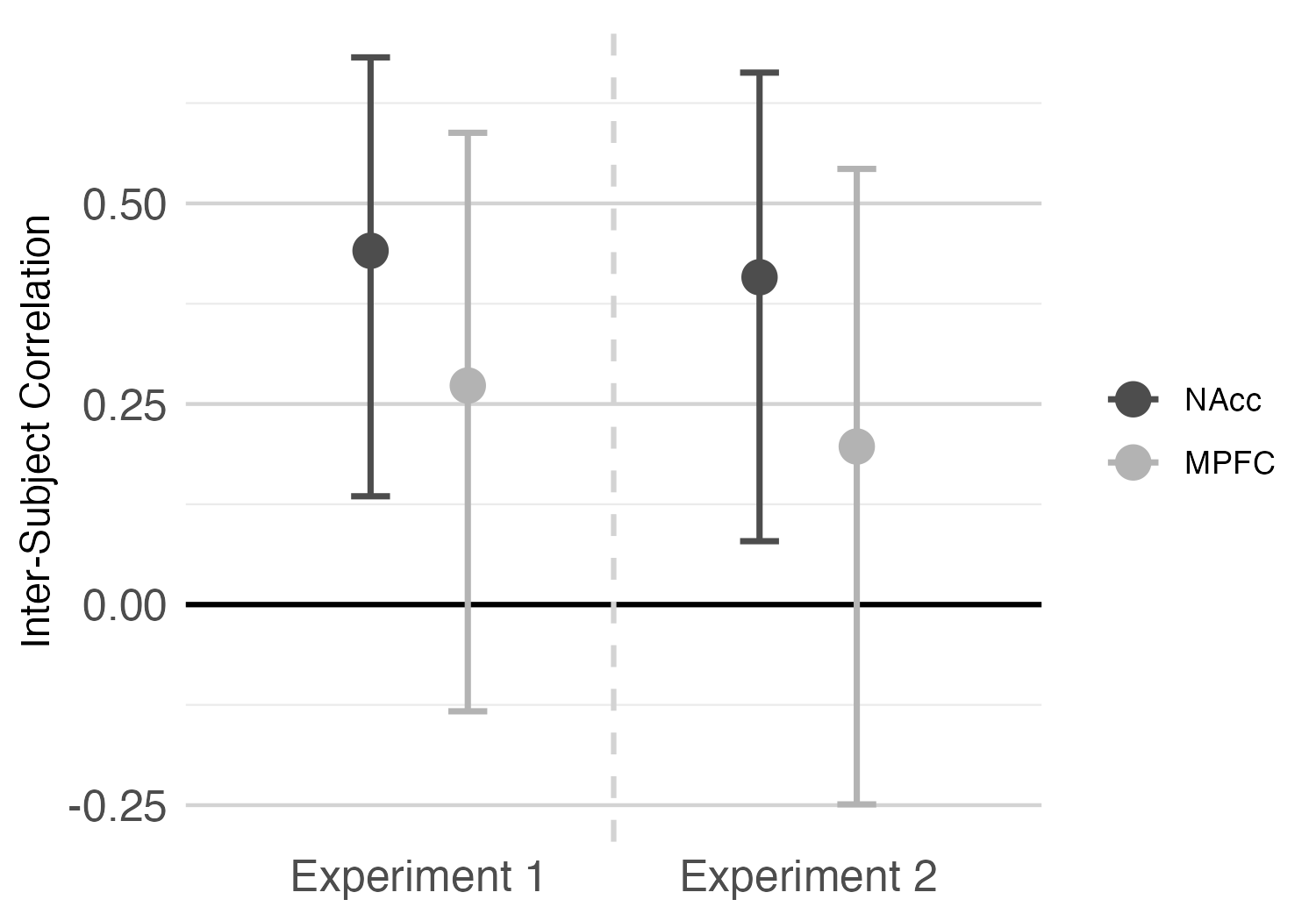


**Fig. S3. Correlations of neural activity across individuals.** Interclass correlation estimates and 95% confidence intervals of NAcc and MPFC activity in response to presented stimuli across individuals within each neuroimaging sample. Across experiments, the NAcc response shows stronger and more significant correlations across individuals than the MPFC response.

Table S1. Crowdfunding experiment (1): Models forecasting aggregate crowdfunding success rate in more versus less representative markets (median split).

|  | **Most Representative** | | | **Least Representative** | | |
| --- | --- | --- | --- | --- | --- | --- |
|  | Behavior | Neural | Combined | Behavior | Neural | Combined |
| Lab Sample Behavior | .374* (.159) |  | .319* (.151) | .249 (.166) |  | .179  (.156) |
| NAcc Activity |  | .639** (.221) | .613** (.211) |  | .671** (.218) | .656** (.217) |
| MPFC Activity |  | -.264 (.221) | -.334 (.213) |  | -.277 (.218) | -.316 (.219) |
| R^2^ | .140 | .233 | .327 | .062 | .257 | .287 |
| AIC | 101.74 | 99.59 | 96.88 | 104.85 | 98.43 | 98.97 |
| Note: | *p<0.05; **p<0.01; ***p<0.001 | | | | | |

NOTE. -- Statistics are standardized coefficients with standard errors in parentheses. NAcc = Nucleus Accumbens; MPFC = Medial PreFrontal Cortex.

Table S2. Video viewing experiment (2): Models of aggregate video viewing in more versus less representative markets (median split)

|  | **Most Representative** | | | **Least Representative** | | |
| --- | --- | --- | --- | --- | --- | --- |
|  | Behavior | Neural | Combined | Behavior | Neural | Combined |
| Lab Sample Behavior | .006 (.183) |  | -.023 (.168) | -.105 (.182) |  | -.145  (.159) |
| Nucleus Accumbens |  | .519** (.180) | .521** (.184) |  | .586** (.173) | .598** (.174) |
| MPFC |  | -.158 (.180) | -.161 (.185) |  | -.231 (.173) | -.251 (.175) |
| R^2^ | .001 | .225 | .226 | .011 | .283 | .304 |
| AIC | 95.79 | 89.64 | 91.62 | 95.44 | 87.16 | 88.22 |
| Note: | *p<0.05; **p<0.01; ***p<0.001 | | | | | |

NOTE. -- Statistics are standardized coefficients with standard errors in parentheses.

Table S3. Models forecasting aggregate choice in (A) crowdfunding and (B) video viewing experiments including the Anterior Insula (AIns) Volume Of Interest.

A)

|  | **Most Representative Quartile** | | | **Least Representative Quartile** | | |
| --- | --- | --- | --- | --- | --- | --- |
|  | Behavior | Neural | Combined | Behavior | Neural | Combined |
| Lab Sample Behavior | 0.441** (0.154) |  | 0.400* (0.148) | 0.275 (0.165) |  | 0.214 (0.159) |
| NAcc Activity |  | 0.563* (0.233) | 0.531* (0.214) |  | 0.671** (0.231) | 0.653** (0.229) |
| MPFC Activity |  | -0.272 (0.256) | -0.360  (0.209) |  | -0.265 (0.224) | –0.312 (0.224) |
| AIns Activity |  | 0.138  (0.168) | 0.138  (0.154) |  | -0.093  (0.168) | -0.093  (0.165) |
| R^2^ | 0.194 | 0.226 | 0.373 | 0.076 | 0.238 | 0.280 |
| Note: | *p<0.05; **p<0.01; ***p<0.001 | | | | | |

*B)*

|  | **Most Representative Quartile** | | | **Least Representative Quartile** | | |
| --- | --- | --- | --- | --- | --- | --- |
|  | Behavior | Neural | Combined | Behavior | Neural | Combined |
| Lab Sample Behavior | 0.065 (0.182) |  | 0.007 (0.174) | -0.189 (0.179) |  | 0.248 (0.161) |
| NAcc Activity |  | 0.597* (0.235) | 0.596* (0.242) |  | 0.685** (0.226) | 0.734** (0.223) |
| MPFC Activity |  | -0.182 (0.211) | -0.182  (0.215) |  | 0.005 (0.203) | –0.009 (0.199) |
| AIns Activity |  | -0.230  (0.268) | -0.229  (0.275) |  | -0.319  (0.258) | -0.373  (0.254) |
| R^2^ | 0.004 | 0.210 | 0.210 | 0.036 | 0.266 | 0.326 |
| Note: | *p<0.05; **p<0.01; ***p<0.001 | | | | | |

**Table S4**. Independent models of aggregate choice in crowdfunding and video viewing experiments for quartiles determined by demographic match

|  | **Crowdfunding experiment** | | | | | | **Video viewing experiment** | | | | |
| --- | --- | --- | --- | --- | --- | --- | --- | --- | --- | --- | --- |
|  | Q1 | | Q2 | | Q3 | Q4 | | Q1 | Q2 | Q3 | Q4 |
| Lab Sample Behavior | .400* (.148) | | .212  (.156) | | .138  (.155) | .214 (.157) | | .027  (.171) | -.040  (.166) | -.087  (.156) | -.216  (.162) |
| Nucleus Accumbens | .579**  (.206) | | .628**  (.218) | | .678**  (.216) | .621** (.219) | | .471*  (.188) | .513**  (.182) | .626**  (.172) | .530**  (.178) |
| MPFC | -.353  (.208) | | -.296  (.220) | | -.307  (.219) | -.317  (.221) | | -.265  (.189) | -.060  (.183) | -.341  (.172) | -.145  (.179) |
| R^2^ | .356 | | .281 | | .291 | .273 | | .189 | .241 | .327 | .272 |
| Note: |  |  | | *p<0.05; **p<0.01; ***p<0.001 | | | | | | | |

NOTE. -- In each experiment, Q1 is the most representative quartile and Q4 is the least representative quartile. Statistics are standardized coefficients with standard errors in parentheses.

**Supporting Text**

**Appendices**

Contents:

Appendix A: Sociodemographic characteristics of subjects in crowdfunding and video viewing experiments

Appendix B: Crowdfunding data collection instruments and stimuli

Appendix C: Video viewing data collection instruments and stimuli

Appendix D: Neuroimaging regions of interest

**Appendix A**

**Sociodemographic characteristics of subjects in crowdfunding and video viewing experiments**

| Baseline characteristic | Crowdfunding Lab (n=37) | | Crowdfunding Online (n=2956) | | Video Viewing Lab (n=40) | | Video Viewing Online (n=992) | |
| --- | --- | --- | --- | --- | --- | --- | --- | --- |
|  | *n* | % | *n* | % | *n* | % | *n* | % |
| Age (mean, sd) | 23.6 (4.8) | | 37.3 (11.7) | | 25.3 (7.4) | | 37.5 (11.0) | |
| Gender |  |  |  |  |  | |  |  |
| Female | 17 | 46 | 1478 | 50.0 | 25 | 62.5 | 661 | 66.6 |
| Male | 20 | 53 | 1478 | 50.0 | 15 | 37.5 | 331 | 33.3 |
| Ethnic Identification |  |  |  |  |  |  |  |  |
| Black or African American | 2 | 5.4 | 266 | 9.0 | 2 | 5.0 | 234 | 23.6 |
| American-Indian / Alaska Native | 1 | 2.7 | 27 | 0.9 | 0 | 0 | 16 | 1.6 |
| Asian | 19 | 51.4 | 205 | 6.9 | 15 | 37.5 | 32 | 3.2 |
| White / European | 10 | 27.0 | 2249 | 76.1 | 13 | 32.5 | 662 | 66.7 |
| Hispanic / Latin | 3 | 8.1 | 159 | 5.4 | 0 | 0 | 40 | 4.0 |
| Other (incl. mixed) | 2 | 5.4 | 50 | 1.7 | 10 | 25.0 | 8 | 0.8 |
| Marital status |  |  |  |  |  |  |  |  |
| Single never married | 32 | 86.5 | 1299 | 43.9 | 28 | 84.8 | 217 | 21.9 |
| Married/partnered | 5 | 13.5 | 1328 | 44.9 | 5 | 15.2 | 715 | 72.1 |
| Divorced | 0 | 0 | 230 | 7.8 | 0 | 0 | 30 | 3.0 |
| Widowed | 0 | 0 | 42 | 1.4 | 0 | 0 | 13 | 1.3 |
| Separated | 0 | 0 | 57 | 1.9 | 0 | 0 | 17 | 1.7 |
| Socio-economic level |  |  |  |  |  |  |  |  |
| Lower | 18 | 48.6 | 439 | 14.6 | 9 | 27.3 | 66 | 6.7 |
| Lower-middle | 5 | 13.5 | 824 | 27.9 | 6 | 18.2 | 147 | 14.8 |
| Middle | 6 | 16.2 | 1395 | 47.2 | 9 | 27.3 | 605 | 61.0 |
| Upper-middle | 4 | 10.8 | 281 | 9.5 | 6 | 18.3 | 163 | 16.4 |
| Upper | 4 | 10.8 | 17 | 0.6 | 3 | 9.1 | 11 | 1.1 |
| Highest educational level |  |  |  |  |  |  |  |  |
| < High school | 0 | 0 | 21 | 0.7 | 0 | 0 | 6 | 0.6 |
| High school graduate | 1 | 2.7 | 294 | 9.9 | 2 | 5.0 | 63 | 6.4 |
| Some college | 13 | 35.1 | 679 | 23.0 | 15 | 37.5 | 83 | 8.4 |
| 2-year degree | 0 | 0 | 352 | 11.9 | 0 | 0 | 47 | 4.7 |
| University degree | 16 | 43.2 | 1164 | 39.4 | 11 | 27.5 | 601 | 60.1 |
| Professional degree | 6 | 16.2 | 394 | 13.3 | 9 | 22.5 | 183 | 18.4 |
| Doctorate | 1 | 2.7 | 52 | 1.8 | 3 | 7.5 | 9 | 0.9 |
| Employment |  |  |  |  |  |  |  |  |
| Employed full-time | 16 | 43.2 | 1945 | 65.8 | 9 | 27.3 | 829 | 83.6 |
| Employed part-time | 0 | 0 | 476 | 16.1 | 3 | 9.1 | 97 | 9.8 |
| Unemployed | 1 | 2.7 | 396 | 13.4 | 2 | 6.1 | 46 | 4.6 |
| Student | 20 | 54.1 | 96 | 3.2 | 19 | 57.6 | 6 | 0.6 |
| Retired | 0 | 0 | 100 | 3.4 | 0 | 0 | 9 | 0.9 |
| Disabled | 0 | 0 | 43 | 1.5 | 0 | 0 | 6 | 0.6 |

**Web Appendix B**

**Crowdfunding experiment data collection instruments and stimuli**

Laboratory FMRI task design. Subjects saw a project image (2 s), project description (6 s), and spatially counterbalanced prompts to indicate their choice to fund or not (4 s), followed by a variable intertrial fixation interval (2– 6 s; adapted from Genevsky et al., (2017)).

Crowdfunding market survey task (kickstarter.com):

In this survey you will see a series of film projects that are seeking funding on an online funding website. You will be asked to decide which film projects you would choose to support.

On each round you will see information about two film projects. You will then select the one that you would prefer to contribute to.

There will be a total of 18 rounds.

After you have made your choices there will be a short survey.

Please try to be as thoughtful and honest with your answers as possible.

When you are ready, press NEXT to begin...

Which of these two film projects would you prefer to contribute to?

[Repeated for 18 trails, for stimuli see below]

Trial (n) of 18

**
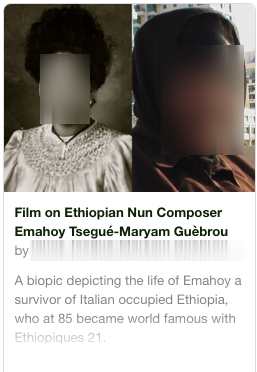

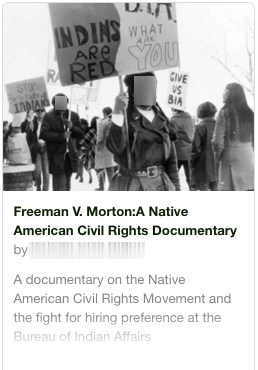
**


Before we end, please tell us just a little about yourself:


Your age

________________________________________________________________

Gender identification

- Male (1)
- Female (2)

Ethnic identification

- Black / African-American (1)
- American-Indian / Alaska Native (2)
- Asian- American (3)
- White / European-American (4)
- Hispanic / Latin-American (5)
- Other (6)

Please select the number that corresponds to your socioeconomic level:

- Lower Income (1)
- Lower Middle Income (2)
- Middle Income (3)
- Upper Middle Income (4)
- Upper Income (5)

Marital status

- Married (1)
- Widowed (2)
- Divorced (3)
- Separated (4)
- Never married (5)

Highest level of formal education achieved

- Less than high school (1)
- High school graduate (2)
- Some college (3)
- 2 year degree (4)
- 4 year degree (5)
- Professional degree (6)
- Doctorate (7)

What is your current employment status?

- Employed full time (1)
- Employed part time (2)
- Unemployed looking for work (3)
- Unemployed not looking for work (4)
- Retired (5)
- Student (6)
- Disabled (7)

Please let us know if you have any had any problems with the survey or if you have any other comments. (optional)

________________________________________________________________

________________________________________________________________

________________________________________________________________

**Experiment 1 stimuli: (faces and names blurred for privacy)**

**
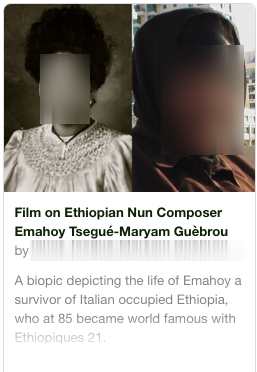

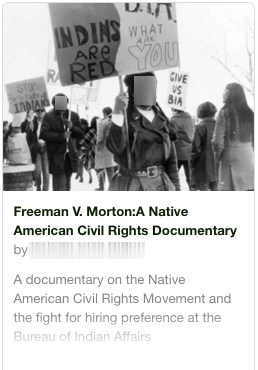

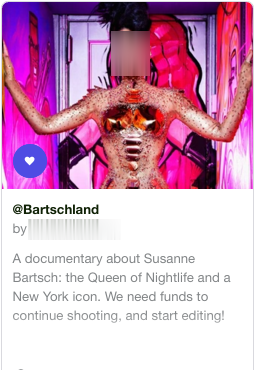

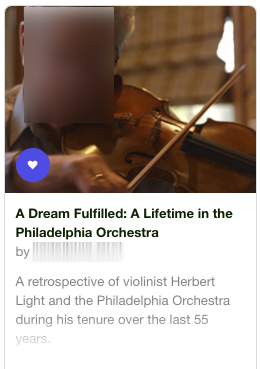

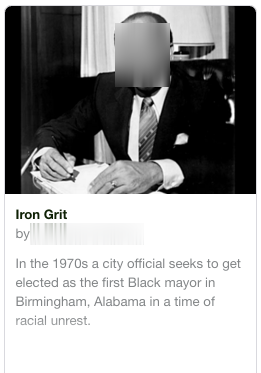

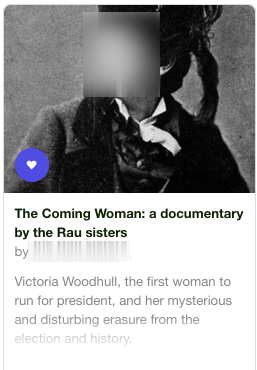

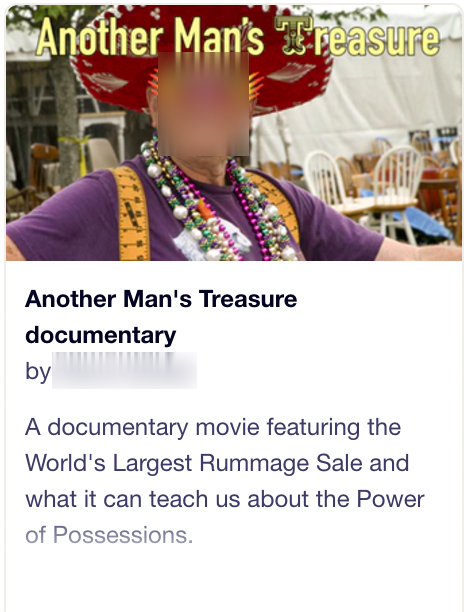

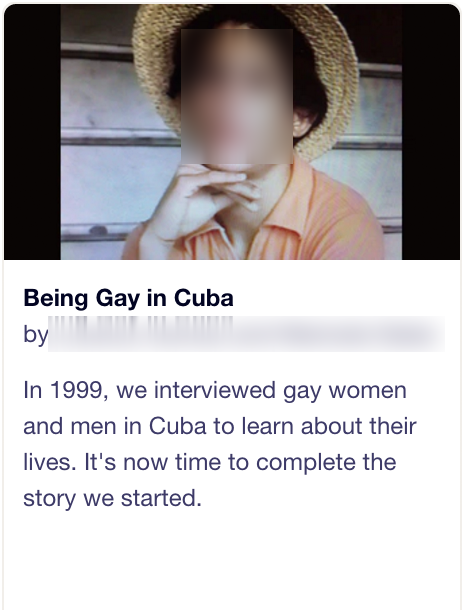

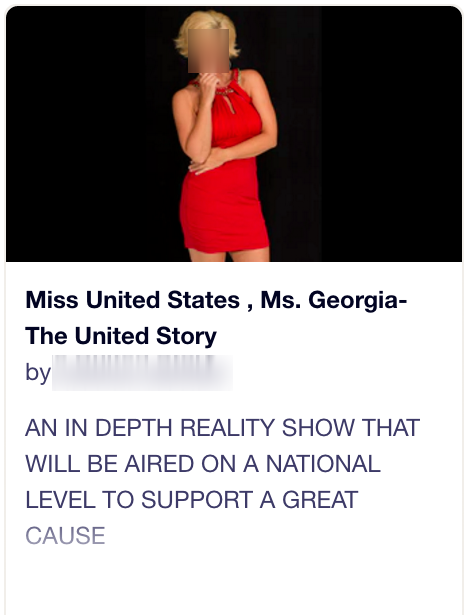

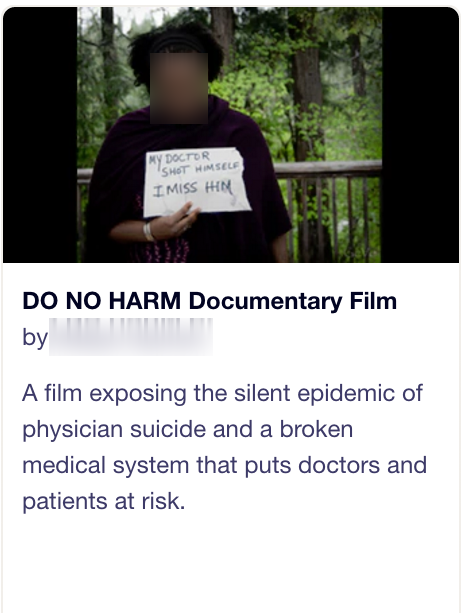

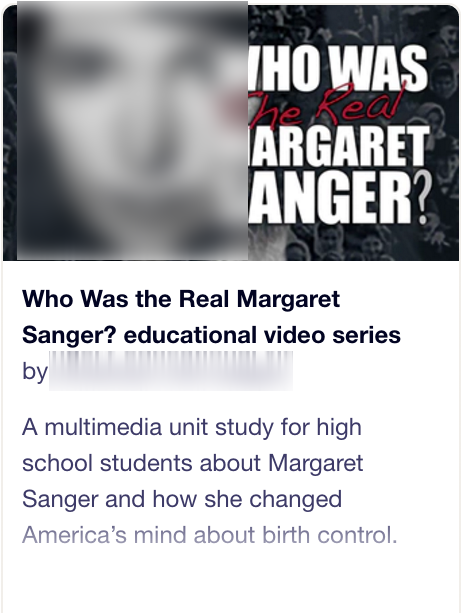

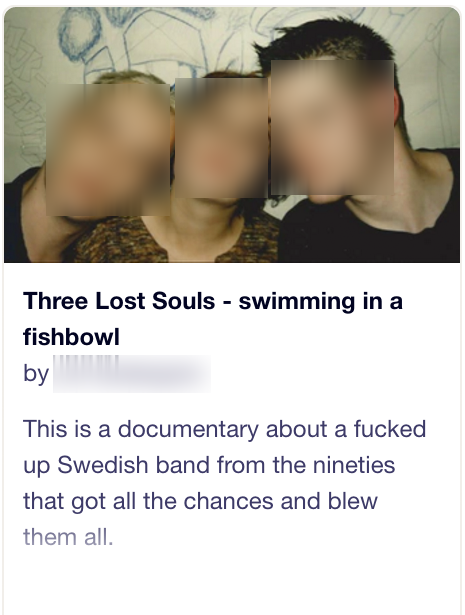

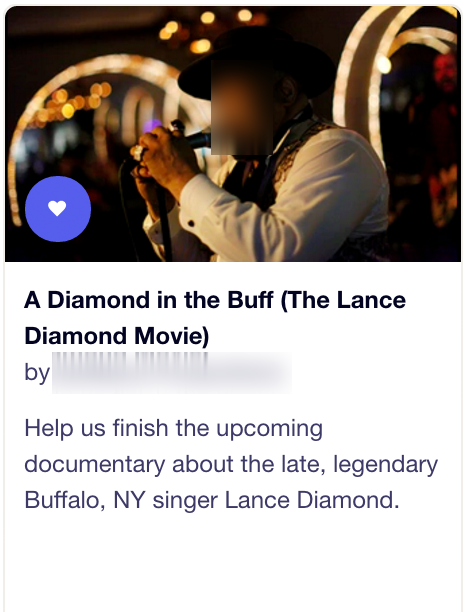

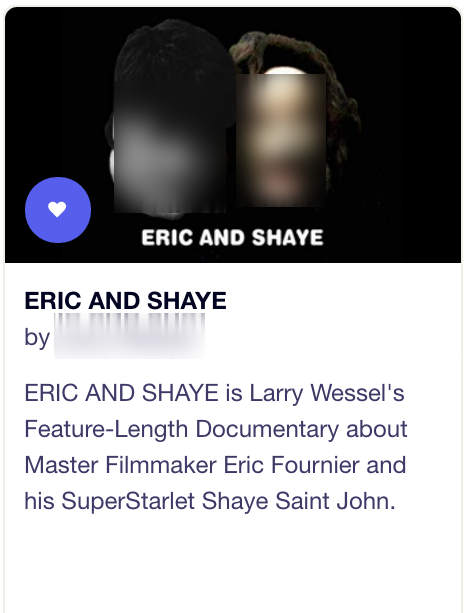

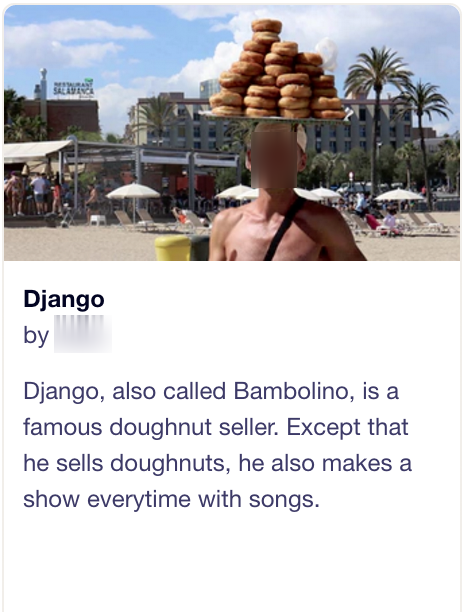

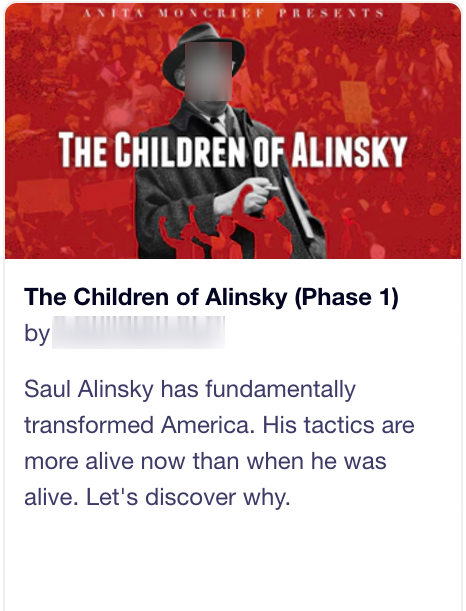

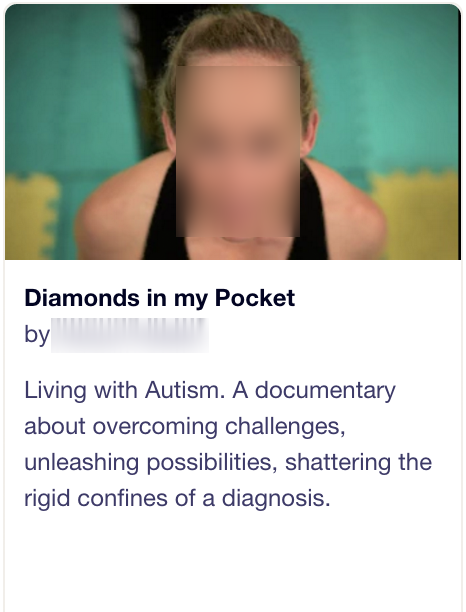

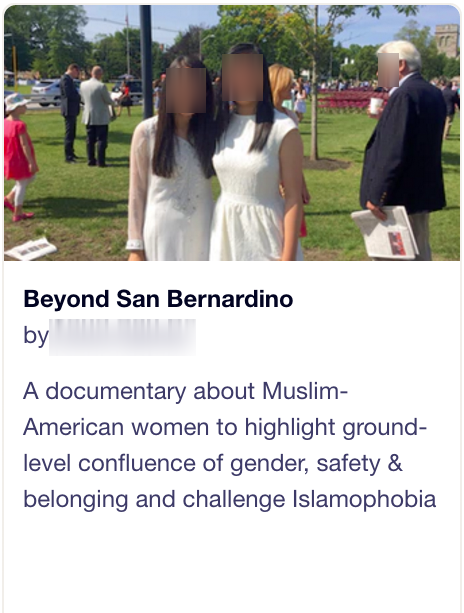

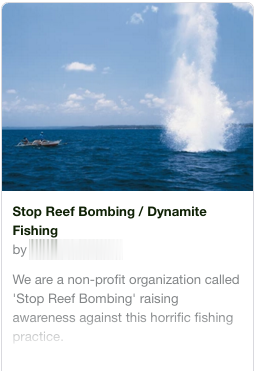

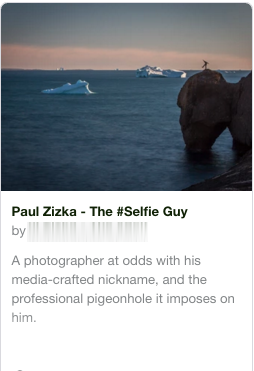

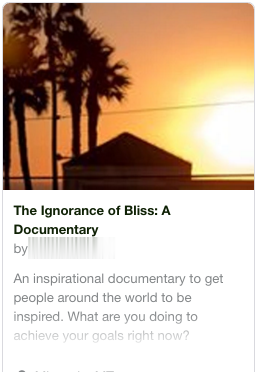

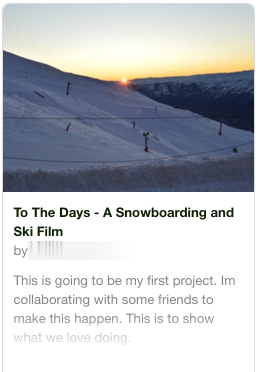

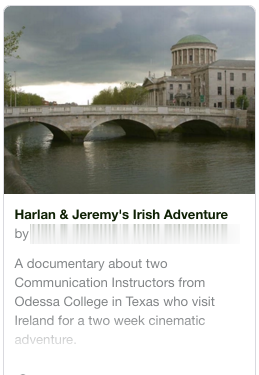

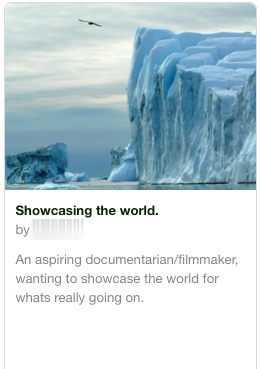

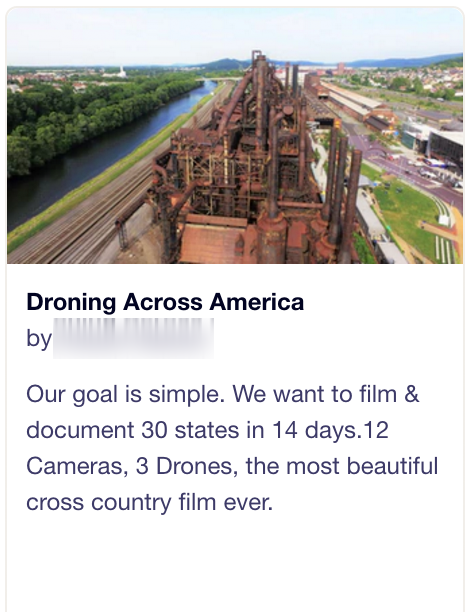

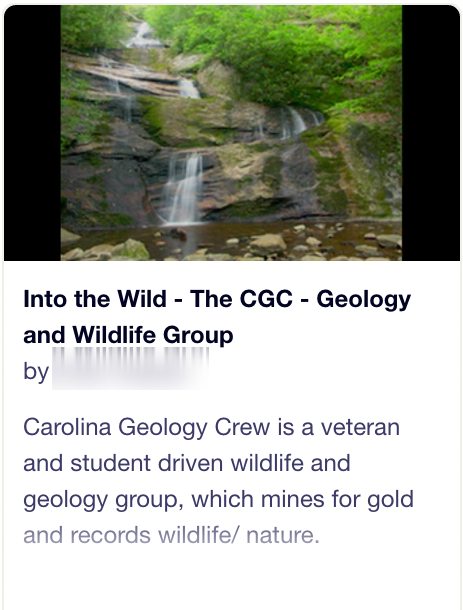

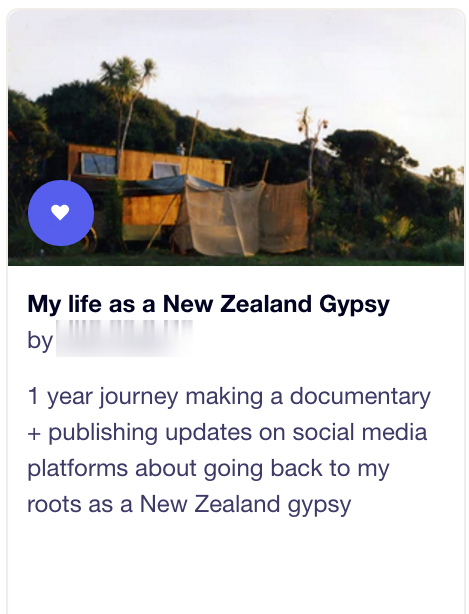

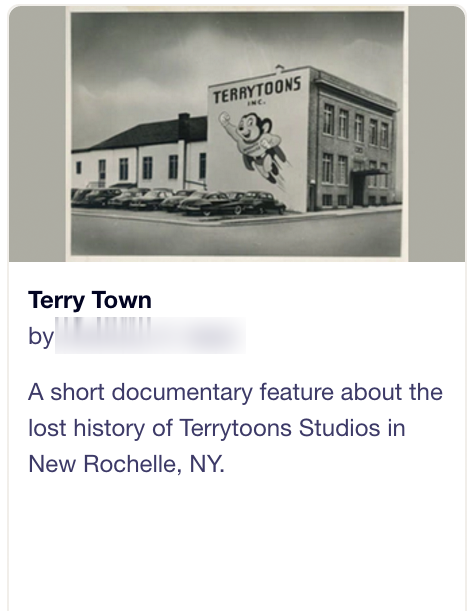

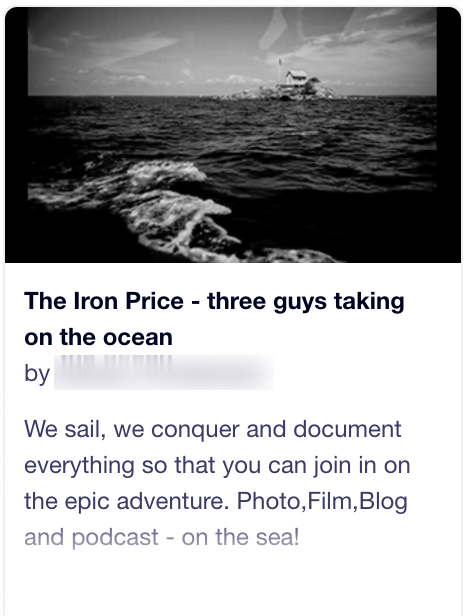

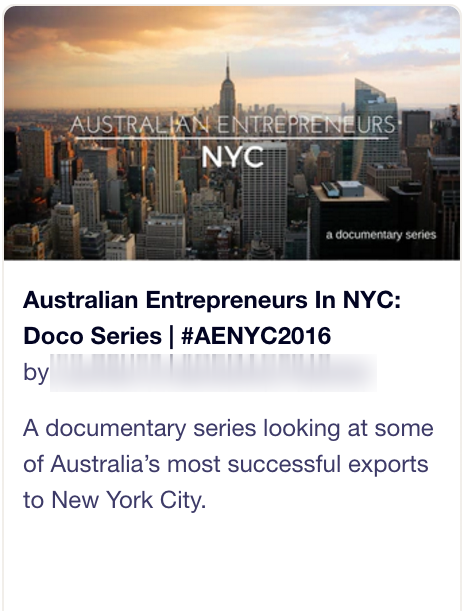

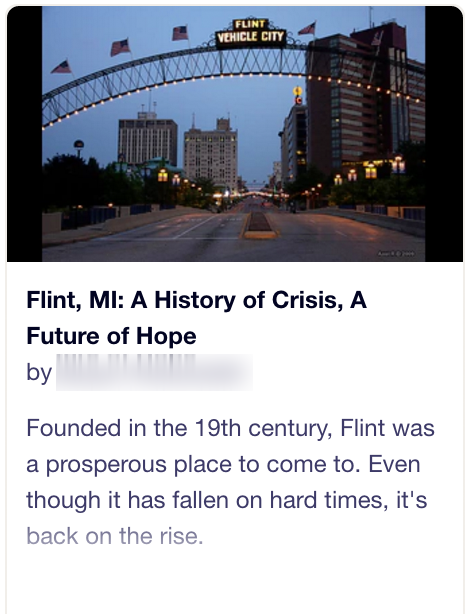

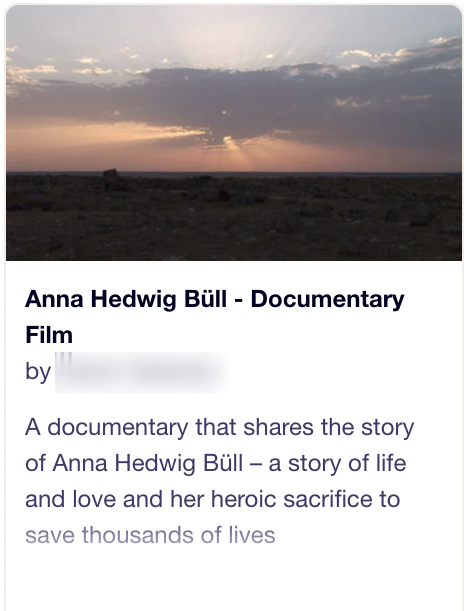

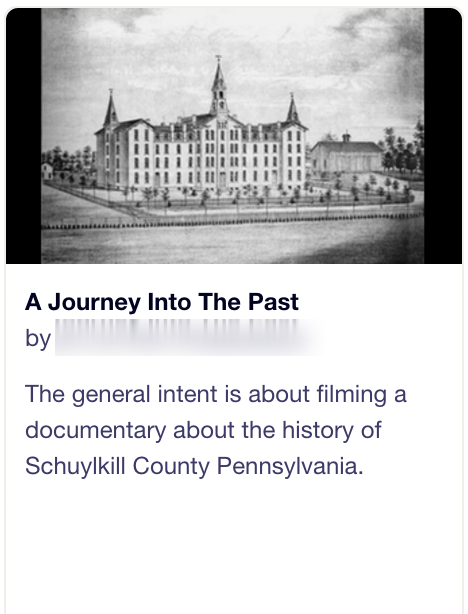

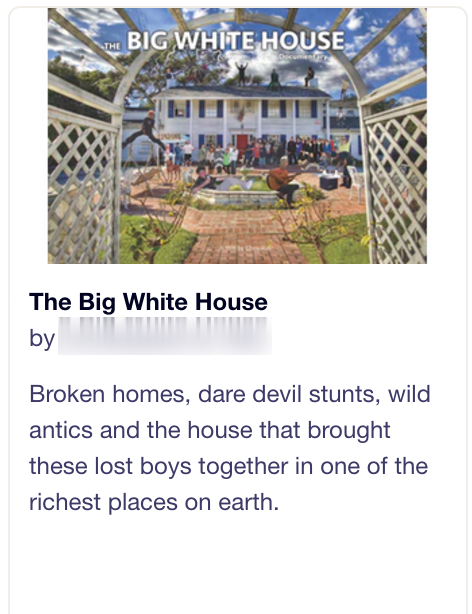

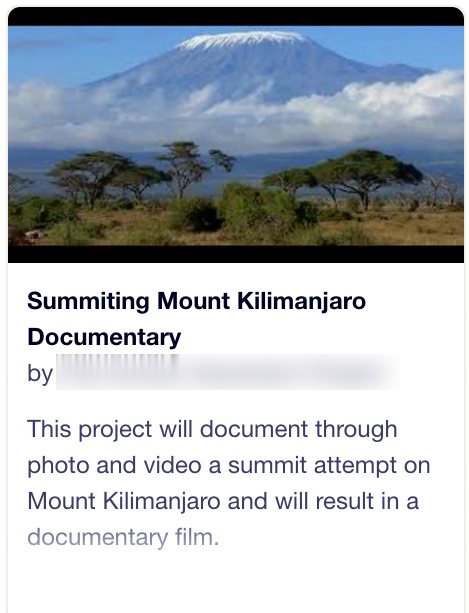

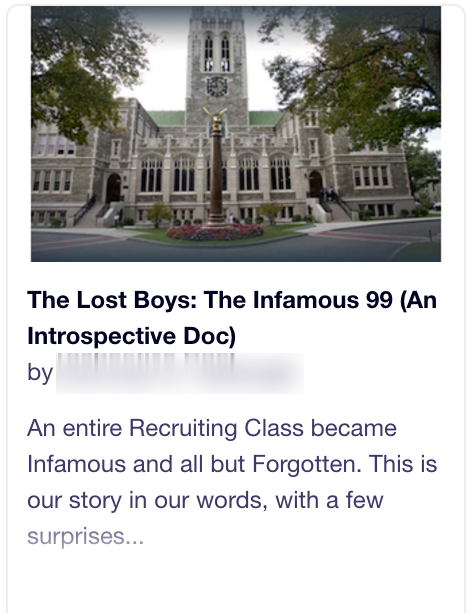
**

**Appendix C**

**Video viewing experiment data collection instruments and stimuli**

Laboratory FMRI task design. Subjects watched each video (4 to 8 s), saw a prompt that allowed them to stop watching (stop), before continuing to watch (or not) and rating the videos (adapted from Tong et al., (2020).

**Video viewing market survey task (youtube.com):**


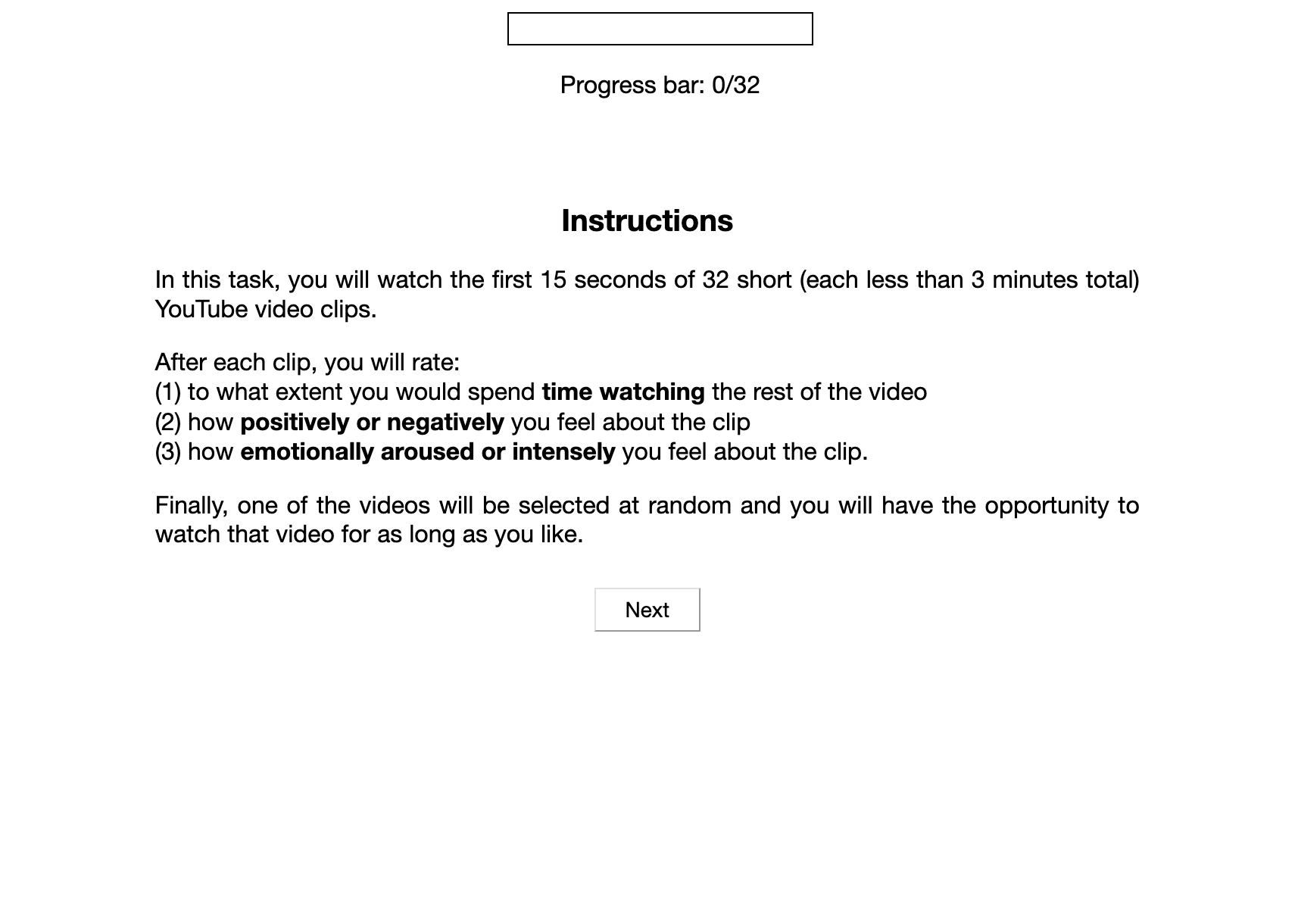


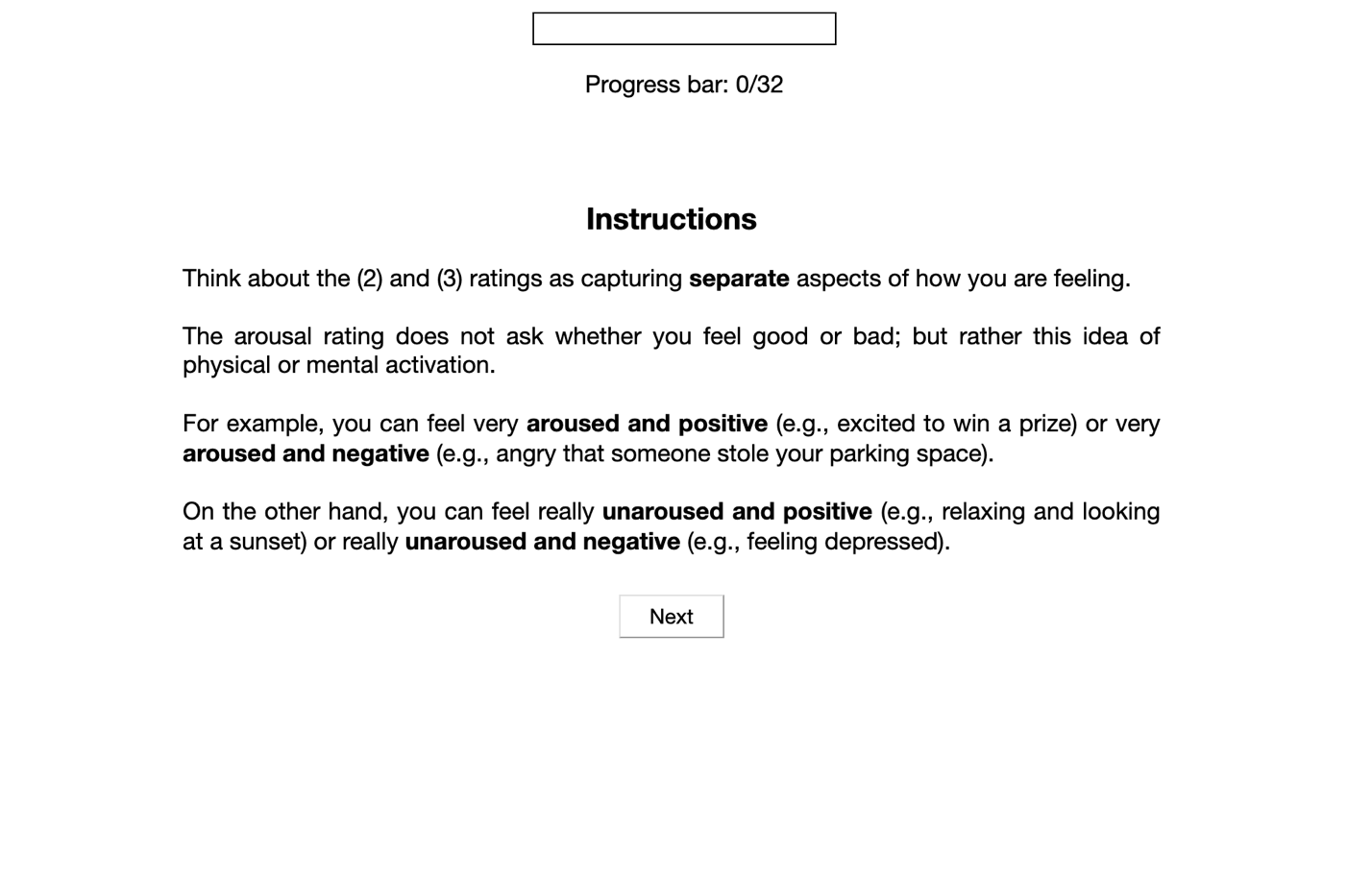


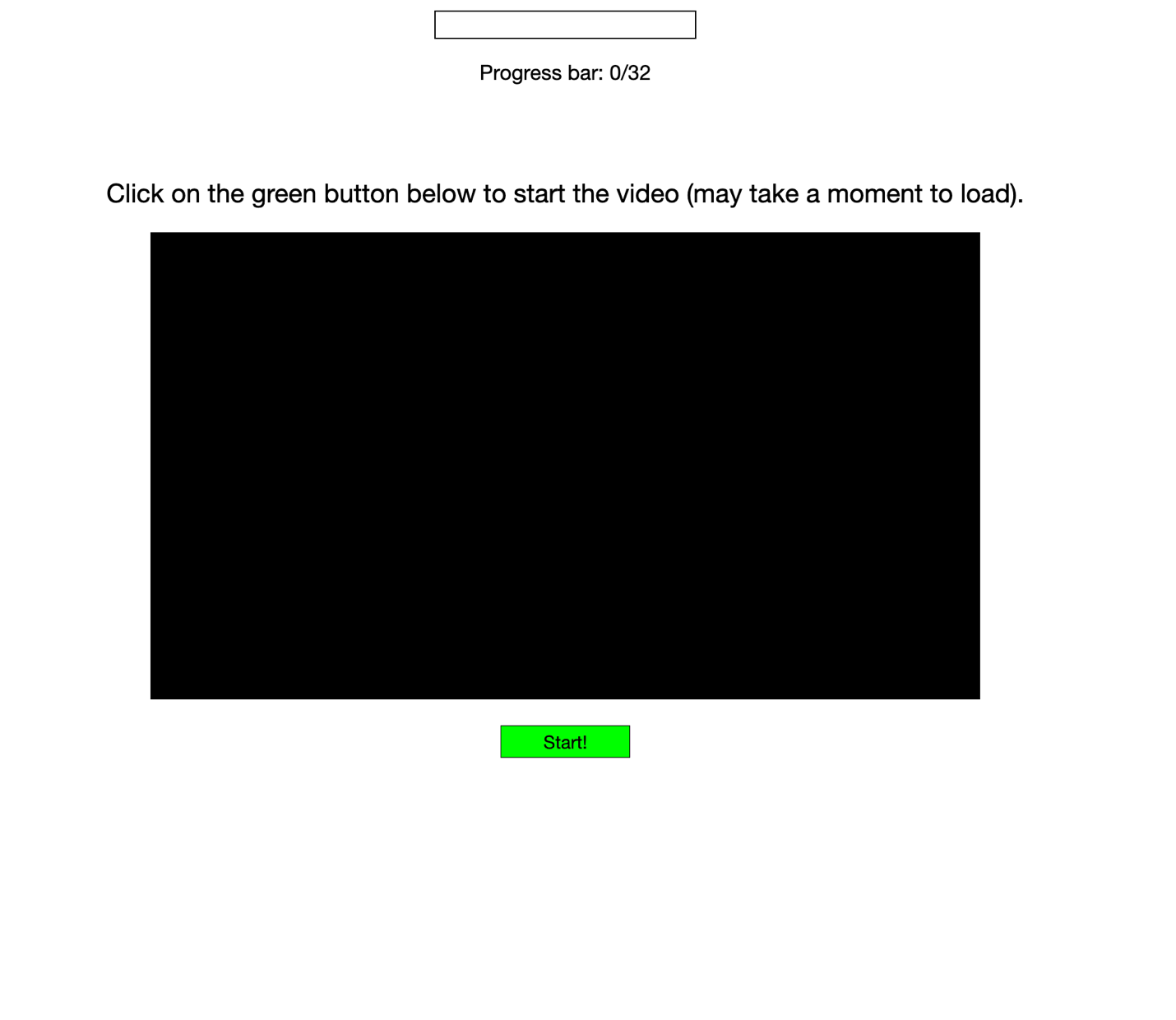

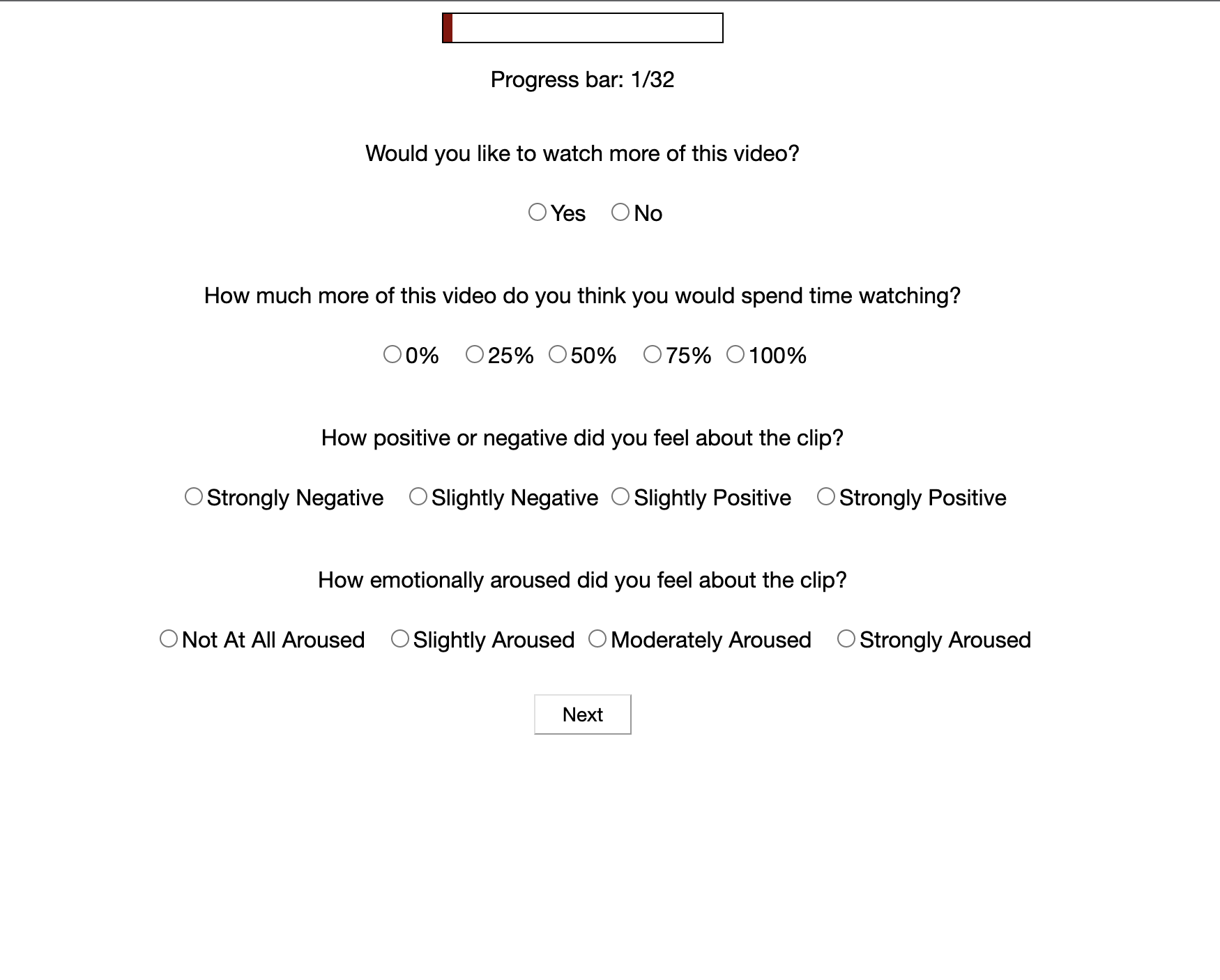


[*repeated for 30 trials*]

Please answer a few final questions:

Your age:

Gender identification:
Male
Female

Ethnic identification
Black / African-American
American-Indian / Alaska Native
Asian-American
White / European-American
Hispanic / Latin-American
Other

Please select your socioeconomic level:
Lower Income
Lower Middle Income
Middle Income
Upper Middle Income
Upper Income

Marital Status:

Married
Widowed
Divorced
Separated
Never married

Highest level of formal education achieved:
Less than high school
High school graduate
Some college
>2 year degree
4 year degree
Professional degree
Doctorate

What is your current employment status?

Employed full time
Employed part time
Unemployed looking for work
Unemployed not looking for work
Retired
Student
Disabled

**List of video stimuli (sourced from youtube.com):**

| Links: |
| --- |
| http://youtube.com/watch?v=uWC6Ioq7_Cc |
| http://youtube.com/watch?v=SMzBBDzT_zY |
| http://youtube.com/watch?v=x9-76dOnHDg |
| http://youtube.com/watch?v=zGeGOJrmqCE |
| http://youtube.com/watch?v=MS7_mYX8Zs8 |
| http://youtube.com/watch?v=h00CuXxeVWQ |
| http://youtube.com/watch?v=jVOmJBnsCD8 |
| http://youtube.com/watch?v=y_pqxpE-eRw |
| http://youtube.com/watch?v=Bhk9WZLJqws |
| http://youtube.com/watch?v=hbXc5nLfzPU |
| http://youtube.com/watch?v=FwNIF7R_wNk |
| http://youtube.com/watch?v=bXuSo88agWc |
| http://youtube.com/watch?v=P_mjA_uanwA |
| http://youtube.com/watch?v=5qUuGkKXDRo |
| http://youtube.com/watch?v=X8wFxwvVriU |
| http://youtube.com/watch?v=LDLzyT-9eJI |
| http://youtube.com/watch?v=x040EqifnN4 |
| http://youtube.com/watch?v=KtexwZeUk7w |
| http://youtube.com/watch?v=5Njo_5VqmDI |
| http://youtube.com/watch?v=diLmCfnsMJ8 |
| http://youtube.com/watch?v=TLk0JWzufGg |
| http://youtube.com/watch?v=R9tQXY2GNxI |
| http://youtube.com/watch?v=xrowWGi20bM |
| http://youtube.com/watch?v=Vppo4jB1o3g |
| http://youtube.com/watch?v=Av-RjQpwAY4 |
| http://youtube.com/watch?v=zpP-tXuuKzQ |
| http://youtube.com/watch?v=5r3llTNTODs |
| http://youtube.com/watch?v=cfGpfMhz-0M |
| http://youtube.com/watch?v=hdXJqfH_unU |
| http://youtube.com/watch?v=w1hHwqOeHN0 |

**Appendix D**

**Neuroimaging regions of interest**

***Overlay of study NAcc and MPFC VOIs (green) on Neurosynth meta-analytic map derived from search term “value” (warm colors)***

Nucleus Accumbens (NAcc). 8mm diameter; coordinates: ±10, 12, -2


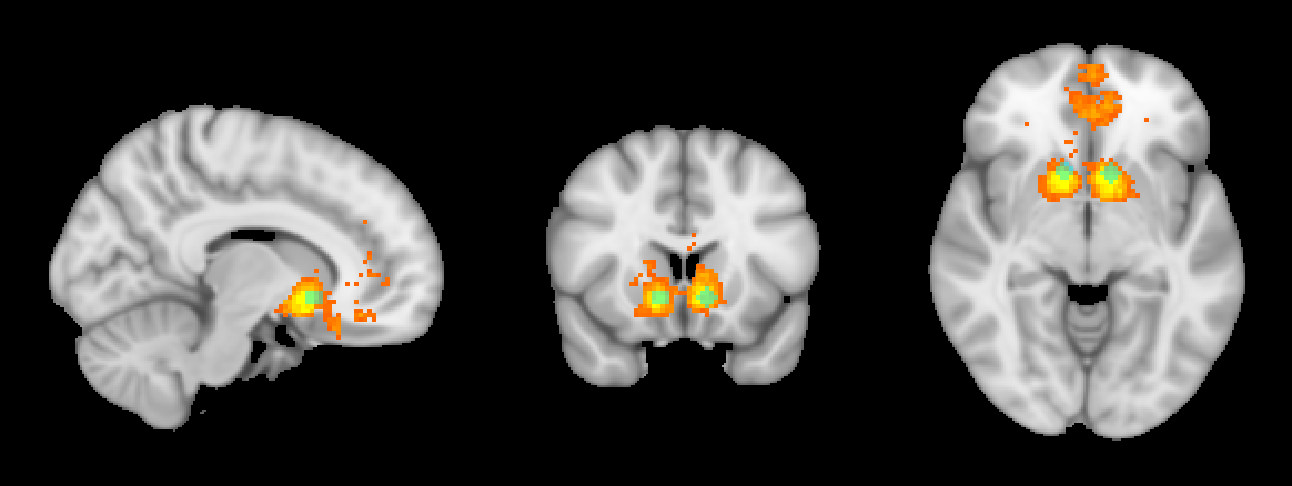


Medial Prefrontal Cortex (MPFC). 8mm diameter; coordinates:  ±4, 45, 0


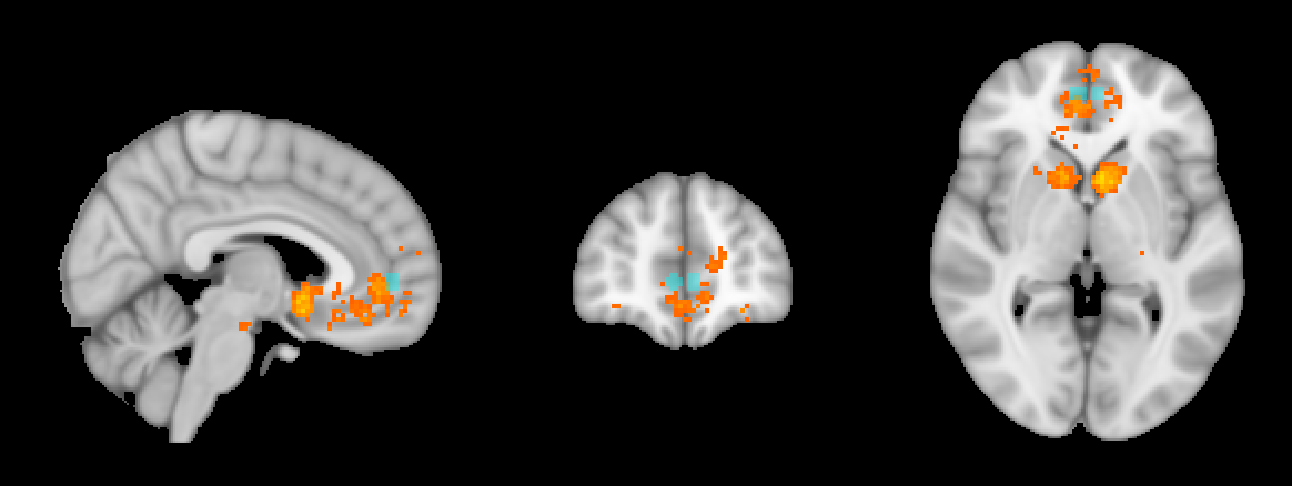


***Overlay of study NAcc VOIs (green) on Neurosynth meta-analytic map derived from the search term “nucleus accumbens”***

Nucleus Accumbens (NAcc). 8mm diameter; coordinates: ±10, 12, -2


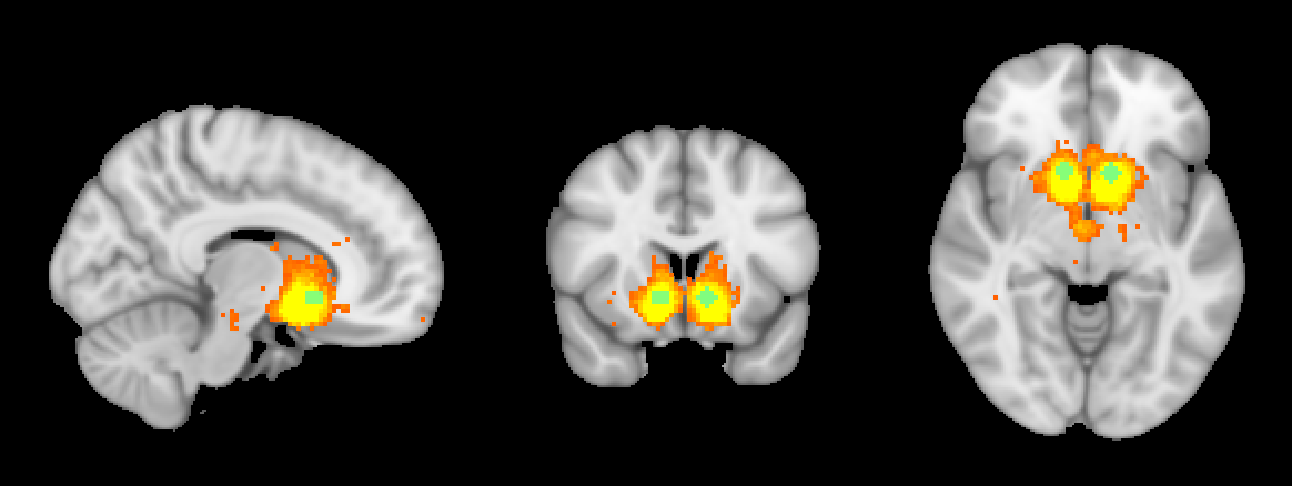


***Overlay of study MPFC VOIs (green) on Neurosynth meta-analytic map derived from the search term “medial prefrontal”***

Medial Prefrontal Cortex (MPFC). 8mm diameter; coordinates:  ±4, 45, 0


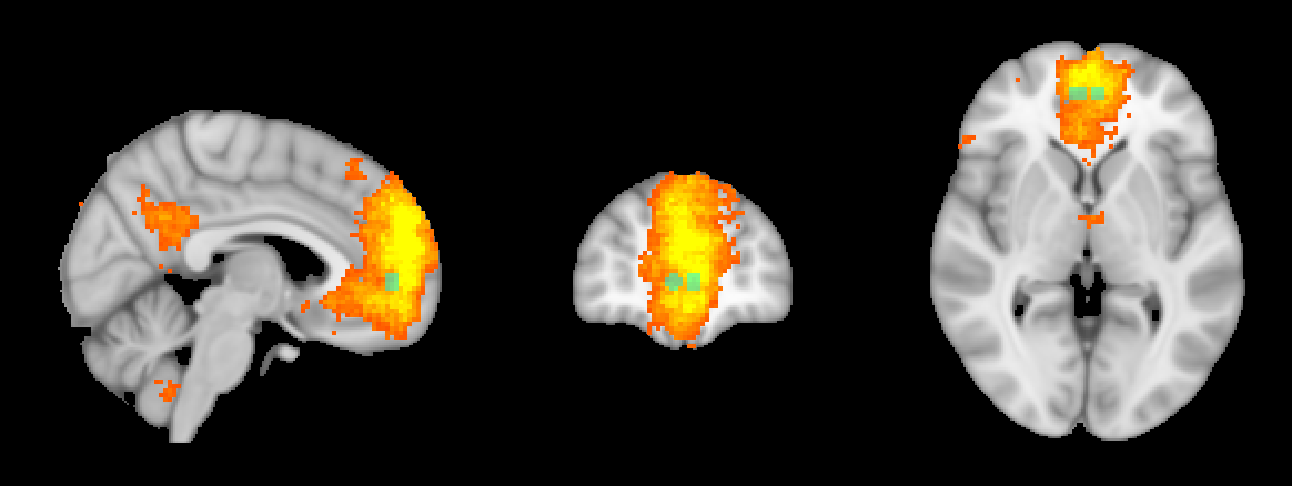

Supplement: pgaf029_Supplementary_Data [file pgaf029_supplementary_data.docx]
